# Spatiotemporal Patterns in Diversity and Assembly Process of Marine Protist Communities of the Changjiang (Yangtze River) Plume and Its Adjacent Waters

**DOI:** 10.3389/fmicb.2020.579290

**Published:** 2020-10-06

**Authors:** Xin Guo, Linnan Wu, Lingfeng Huang

**Affiliations:** Key Laboratory of the Ministry of Education for Coastal and Wetland Ecosystems, College of the Environment and Ecology, Xiamen University, Xiamen, China

**Keywords:** protist, diversity, biogeography, community assembly, Changjiang (Yangtze River) plume

## Abstract

Marine protists are highly heterogeneous and play key roles in the structure and functioning of marine ecosystems. However, little is known on the underlying biogeographic processes and seasonal diversity patterns that shape their community assembly in a regional scale in marginal sea. In this study, we conducted high-throughput sequencing of 18S rRNA gene to survey the protist community compositions (PCCs) of the Changjiang (Yangtze River) plume, an intensely human-affected coastal area, to the adjacent continental shelf waters over three seasons. Furthermore, the different impacts of environmental and spatial factors on marine PCCs were examined. The results revealed significant dissimilarities of PCC’s diversity among seasons and habitats, with more obvious seasonal variations in the Changjiang plume. Procrustes analysis showed better consistency of the community-environment relationship in shelf area, further supported by stronger correlation coefficients computed in the Mantel tests. This might be explained by seasonal dynamics of Changjiang Diluted Waters (i.e., the mixing of the Changjiang runoff with inshore water of the East China Sea) that changed the environmental conditions of coastal area dramatically, resulting in lower importance of spatial factors (dispersal limitation) on PCCs compared with environmental filters, including physicochemical properties (e.g., water temperature, salinity, dissolved oxygen, and nutrients), as well as biotic factors (e.g., Chl *a* and food abundance). This was further explained by higher immigration rate and fitness to neutral model, which suggested a predominant role of neutral process in shaping the PCCs in coastal area. Different richness, diversity, and taxonomic compositions but comparable biogeographic patterns were observed among abundant and rare sub-communities. In general, the abundant sub-communities exhibited higher dispersal ability which tend to respond to environmental selection during dispersal, whereas the rare sub-communities appeared to be present only in few samples due to dispersal limitation. Co-occurrence network further indicated the importance of biotic interactions in community assembly and potential roles of rare taxa in maintaining the community structure. Overall, this study suggests the dynamic in the biogeographic patterns of PCCs of the Changjiang plume to the adjacent waters in the ECS responding with the changing environmental conditions and geographical factors.

## Introduction

Protists (single-celled eukaryotes) are significant components in global marine ecosystems with wide distributions and high biodiversity in multiple trophic modes, which play fundamental roles in numerous essential ecological and biogeochemical processes (Adl et al., 2012; Caron et al., 2012; Fuhrman et al., 2015; Keeling and del Campo, 2017). With the application of high-throughput sequencing (HTS) combined with metabarcoding, many studies have investigated the diversity and biogeographic distributions of the protist community compositions (PCCs) under dynamic temporal scales and different habitats (Massana et al., 2015; Marquardt et al., 2016; Pernice et al., 2016; Wu et al., 2016; Liu et al., 2017; Xue et al., 2018; Zhang W. J. et al., 2018; Xu et al., 2020). However, much fewer studies have focused on protist community compared with bacterial communities (Keeling and del Campo, 2017).

Metacommunity theory has been applied to address the underlying mechanisms of community assembly for many natural communities (Leibold et al., 2004; Cottenie, 2005). Specifically, two categories of processes have been used to explain the biogeographic distribution patterns (Dini-Andreote et al., 2015). The first one is the deterministic process supported by niche theory, emphasizing the dominant role of environmental factors that have direct or indirect impacts on microbial communities and result in niche differentiation (Keddy, 1992; Caron and Hutchins, 2012). The other category is the stochastic process supported by neutral theory (Hubbell, 2006), suggesting that the biogeographic distribution patterns of the community are affected by random processes such as dispersal effect restricted by spatial distance (known as dispersal limitation), ecological or genetic drift, and accidental or historical events (Martiny et al., 2006; Hanson et al., 2012). Further, time and space are the two major scales over which the microbial community composition changes in response to heterogeneous environment and at different spatial scales (Fuhrman et al., 2015; Langenheder and Lindström, 2019). Specifically, it is likely that the metacommunity might be driven by environmental filtering during certain periods and by dispersal during other periods within the same ecosystem (Gilbert et al., 2012; Shade et al., 2014; Yeh et al., 2015; Wu et al., 2016; Nino-Garcia et al., 2017) because the degrees of environmental heterogeneity and spatial connectivity change over time (Yeh et al., 2015). For the spatial scale, it is commonly agreed that the importance of spatial factors increases in a larger spatial range due to dispersal limitation (Hanson et al., 2012). However, the importance of environmental factors can also overwhelm the spatial factors due to strong environmental heterogeneity even at a continental scale (Zhang W. J. et al., 2018). In summary, both the environmental and spatial factors should be considered when determining the PCCs assembly in different time and space scales.

Different taxonomic lineages may be assembled differently in abundance due to their intrinsic properties (Logares et al., 2015; Nino-Garcia et al., 2016b, 2017), and thus cause comparable properties in biogeographic processes and patterns (Wu et al., 2016). Previous studies mostly focused on a few high-abundance taxa, while ignoring the low abundant or rare taxa with strikingly disproportionate high diversity in the environment (Caron and Countway, 2009; Galand et al., 2009; Shade et al., 2014; Logares et al., 2015). With the rapid development of HTS technology and increasing sequencing depth, an increasing number of rare taxa are discovered (Caron et al., 2012; Logares et al., 2014; Lynch and Neufeld, 2015). Moreover, the processes that drive community assembly of rare taxa compared with the abundant taxa have attracted increasing attention. For example, “temporally rare taxa,” which are constrained in some periods by the environment or other specific dispersal limitations, may modify the whole community under certain conditions (Shade et al., 2014; Lynch and Neufeld, 2015; Shade and Gilbert, 2015). Rare taxa can also have ubiquitous or restricted spatial distributions (Nino-Garcia et al., 2016b) and may show clear seasonal patterns and abundance peaks over time (Nino-Garcia et al., 2017). Thus, further studies are needed to investigate the community structures of both abundant and rare sub-communities to trace their responses to biogeographical processes and to further explore their ecological functions.

The East China Sea (ECS) is a marginal sea with sloping topography (Ichikawa and Beardsley, 2002), leading to a wide coastal area and an adjacent open continental shelf area. Among this, the region of Changjiang (Yangtze River) plume and the adjacent waters is one of the most complex and dynamic systems for the study of the PCCs’ diversity and contributions. Multiple water masses from different directions affect the ecological environment of this area (Ichikawa and Beardsley, 2002; Lee and Chao, 2003). Moreover, seasonally changing circulations have caused the currents systems more complex (Ichikawa and Beardsley, 2002; Lee and Chao, 2003). Specifically, the dispersal of Changjiang Diluted Waters (CDW) gradually expands outwards with the increase of the Changjiang runoff from spring to summer and the horizontal gradient of salinity increases (Ichikawa and Beardsley, 2002; Lee and Chao, 2003). Moreover, the Taiwan Warm Currents intrusion from the south is strong in summer (Li et al., 2014). Thus, the high temperature and salinity seawaters in the shelf area that come from Kuroshio currents retreat southward. In autumn, the runoff of CDW is greatly weakened and turns to the South along the coast, forming a long and narrow freshwater belt along the Jiangsu and Zhejiang coasts, while the high salinity water in the open shelf sea is greatly strengthened and expands northward (Lee and Chao, 2003). Besides, previous studies reported high nutrient input and hypoxia of the Changjiang plume area that had impact on the microbial communities (Guo et al., 2014; Liu et al., 2017; Zhou et al., 2020), which was affected strongly by anthropogenic activities compared with peripheral continental shelf area (Li and Daler, 2004). Dispersal-related stochastic processes caused by current transportation could also play critical roles in regulating the microbial communities (Xu et al., 2020). Thus, the hydrological conditions display highly different spatial and temporal patterns between the coastal areas of Changjiang plume and its adjacent shelf areas (Yeh et al., 2015; Liu et al., 2017), which should affect community assembly of PCCs differently, but is so far understudied. In summary, the region of Changjiang plume and the adjacent shelf waters provides a suitable system for the study of the relative importance of regional environmental conditions and dispersal processes for determining PCCs’ metacommunity in different habitats over time.

Using high-throughput sequencing of 18S rRNA gene V4 region, this study investigates the spatiotemporal dynamics of PCCs of the Changjiang plume and the adjacent shelf waters in the ECS in three seasonally representative months (i.e., May, August, and October represented spring, summer, and autumn, respectively) of 2013. Furthermore, six categories of protist sub-communities were defined based on their relative abundance and frequencies across all samples. Lastly, we employed Mantel tests, neutral models, and co-occurrence analyses to reveal and explain the changing patterns of biogeographical processes that shape and structure PCCs. The hypotheses of our study were (1) the spatiotemporal patterns of PCCs’ diversity and biogeographic distributions differed in the coastal area of Changjiang plume and the adjacent shelf waters in different seasons, with comparable properties of the abundant and rare sub-communities; (2) environmental factors may have contributed more in the PCCs’ assembly in the studied area due to highly heterogeneous and seasonally dynamic environmental conditions such as temperature, salinity, and nutrient; (3) the current transportation of CDW may weaken the importance of spatial factors (representing dispersal limitation) on PCCs and resulted in high dispersal in the coastal area of Changjiang plume; and (4) other factors, e.g., biological interactions within communities, play important roles in shaping the structure of PCCs.

## Materials and Methods

### Study Area and Sampling

**Figure 1:**
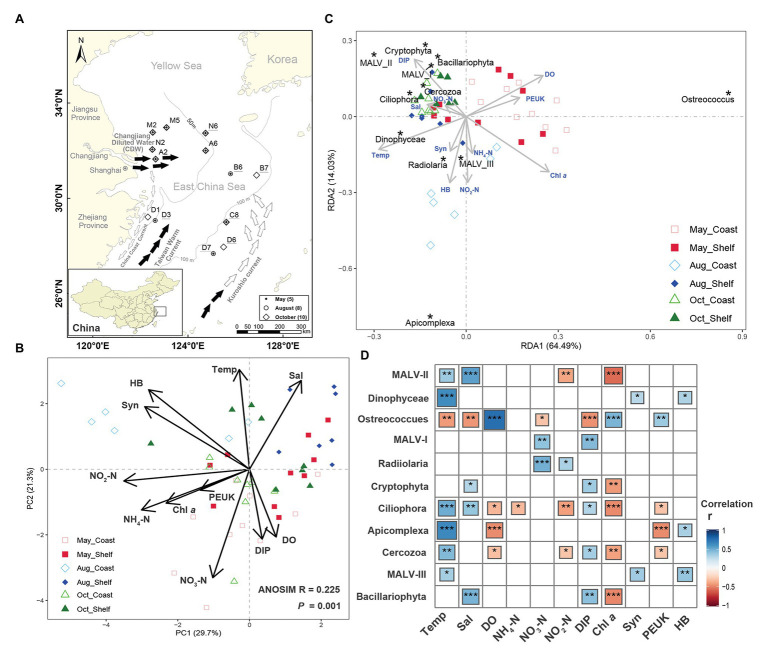
Study area, spatiotemporal variation in environmental conditions, and the relationship with protist dominant lineages across three seasonally representative months and two habitats. **(A)** Location of the 13 sampling stations in the studied area in 3 months in 2013. Water samples in each station were simultaneously collected from both surface and bottom layers. Symbols indicate sampling months. Sample labels with underline indicate stations in shelf area (deeper than 50 m), whereas others represent stations in coastal area (lower than 50 m). The summer currents are indicated with black arrows, whereas white arrows represent spring and autumn currents. **(B)** Ordination biplots of the principal component analysis (PCA) of all environmental variables. **(C)** Redundancy analysis based on 37 lineages of protist and the environmental variables. Only 11 dominant taxa (percentage of reads >1% in all 60 samples) were marked in the figure. **(D)** Correlation analyses among the environmental factors and the relative abundance of the 11 dominant lineages (percentage of reads >1% in all 60 samples). Asterisks inside the squares indicate significant correlations (^***^p < 0.001; ^**^p < 0.01; and ^*^p < 0.05). Environmental codes are as follows: Temp, temperature (°C); Sal, salinity (psu); DO, dissolved oxygen (mg/L); NH_4_-N, ammonia nitrogen (μmol/L); NO_3_-N, nitrate (μmol/L); NO_2_-N, nitrite (μmol/L); DIP, dissolved inorganic phosphorus (μmol/L); Chl *a*, chlorophyll *a* (μg/ml); Syn, *Synechococcus* (cells/ml); PEUK, pico-eukaryotes (cells/ml); HB, heterotrophic bacteria (cells/ml).

Sampling was conducted on board R/V “*Beidou*” (from May 6 to May 21 and from October 10 to November 11, 2013) and R/V “*Dongfanghong 2*” (from August 4 to August 31, 2013). Overall, a total of 10 studied sites were analyzed, as 6 of the 13 sampled stations were geographically close and were assumed to be representative of the same site (i.e., B6 and B7, D6 and D7, and D1 and D3; Figure 1A). A total of 60 samples were collected at two depths (2 m beneath the sea surface as the surface layer and 3–4 m above the seabed as the bottom layer) from each studied site across three sampling months, spanning 27.6–32.8°N, 122–127°E of the Changjiang plume and the adjacent waters in the ECS (Figure 1A and Supplementary Table S1). To simplify the statement of two habitats in this study, the region of Changjiang plume was identified as coastal area as the water depth is shallower than 50 m, whereas the stations with the water depth ranging between 50 and 110 m were identified as shelf area (Figure 1A). Thus, according to the spatiotemporal properties, all the samples were predefined and clustered artificially into 6 month-habitat groups to be compared in subsequent analyses, which were May-coast, May-shelf, August-coast, August-shelf, October-coast, and October-shelf. For protist community analyses, 1.5–2-L seawater samples were pre-filtered through 20 μm nylon mesh by gravity and were then filtered through 0.22-μm polycarbonate filters (47 mm diameter; Millipore, Billerica, MA, United States). Filters were immediately frozen in liquid nitrogen in the field and stored at −20°C until further processing.

### Environmental Abiotic and Biotic Factors

Water temperature, salinity, and depth profiles were recorded *in situ* with a Seabird 19 CTD (SBE917plus; SeaBird, Bellevue, WA, United States) and the concentration of dissolved oxygen (DO) was measured using the traditional Winkler iodometric titration method (Bryan et al., 1976). Samples (100 ml) for nutrients were filtered through Whatman GF/F filters and analyzed by segmented flow automated colorimetry using the manufacturer’s standard procedures (San + + Automated Wet Chemistry Analyzer, The Netherlands). Samples (1 L) for chlorophyll a (Chl *a*) were pre-filtered through a 200-μm-mesh sieve to remove large zooplankton and debris. Filtered water was then passed through 0.7-μm GF/F filters (Whatman, England), with the filters then wrapped in aluminum foil and stored at −20°C, in the darkness. Chl *a* was then extracted by 10 ml of 90% acetone at 0°C for 20 h in the dark and measured using a Trilogy Fluorometer (Turner Designs, Trilogy Module: CHL-A NA). The abundance of heterotrophic bacteria (HB), *Synechococcus* (Syn), and pico-sized eukaryotes (PEUK) were run with a FACSVantage SE flow cytometer (Becton, Dickinson and Company, Franklin Lakes, NJ, United States) equipped with a water-cooled argon laser (488 nm, 13–20 mW, coherent; Zhao et al., 2013).

The sampling and enumeration of nano-sized protist (NP) cells followed the protocol of Lin et al. (2013) using steps of fixing, filtering, staining, and mounting. Specifically, the pre-filtered samples through 20-μm nylon mesh were fixed with cold glutaraldehyde [final concentration 0.5% (v/v)] and then filtered onto a 0.22-μm-pore-size black polycarbonate membrane filter (25 mm in diameter; Millipore, MA, United States) under low vacuum pressure (<100 mm Hg). The samples were then stained with DAPI (final concentration 10 μg/ml) for 7–8 min when only 1 ml was left in the funnel. After completely filtered, the membrane was mounted onto a microscope slide and a cover slip on top of it with a few drops of immersion oil (Sigma-Aldrich, MO, United States) to fix it. Different trophic modes [non-pigmented heterotrophic nano-sized protist (HNP) and pigmented nano-sized protist (PNP)] and three size categories (2–5, 5–10, and 10–20 μm) of NP were grouped and identified according to their fluorescence (Tsai et al., 2005) and cell length (Lin et al., 2013) under epifluorescence microscopy (Leica DM 4500B) at ×1,000 magnification. In short, all cells that meet the fluorescent conditions and size fractions were counted (i.e., those that appeared blue under UV light were the biological cells; pigmented PNPs were distinguished from HNPs by the presence of red autofluorescence under a blue excitation light; and cells that are smaller than 2 μm or larger than 20 μm were discarded). At least 40 fields of view with more than 100 cells (PNP plus HNP) were examined for each filter to obtain reliable estimates of abundance. The significance of difference in the distribution of size structure and trophic structure was analyzed using one-way ANOVA, followed by Duncan’s means comparisons on samples grouped by months and sites.

### DNA Extraction, PCR Amplification, and High-Throughput Sequencing

DNA was extracted from cells collected onto filters that were cut into pieces using the E.Z.N.A. Water DNA Kit (Omega Bio-tek, Norcross, GA, United States) according to the manufacturer’s instructions. DNA extracts were eluted in 50-μl elution buffers provided in the kit, quantified using a Nanodrop ND-2000 Spectrophotometer (Thermo Scientific), and verified on a 1.5% agarose gel. DNA extracts were kept at −20°C until processing. The hypervariable V4 region (ca. 380 bp) of the eukaryotic 18S ribosomal RNA (rRNA) gene was amplified using the primers TAReuk454FWD1 (5'-CCAGCASCYGCGGTAATTCC-3') and TAReukREV3 (5'-ACTTTCGTTCTTGATYRA-3'; Stoeck et al., 2010). Forward and reverse primers were tagged with 2 bp links and 8 bp length of barcodes to allow pooling multiple samples in one run of sequencing and later differentiation of PCR products from different samples (Kozich et al., 2013). Six individual PCR reactions were run per sample to reduce the amplification error and then purified by a PCR Product Recovery kit (BioTeke, Beijing, China), followed by pooling of individual amplicon libraries in equimolar concentrations. The mixture of products was submitted to a commercial sequencing company (BGI group, Guangdong, China) and was run on one lane of a paired-end 2 × 250 bp sequencing run with V3 chemistry on an Illumina Miseq platform.

### Sequence Analyses

A series of filters on raw sequence data were processed to retain only high-quality sequences with MOTHUR v.1.40.5 (Schloss et al., 2009) based on the standard operating procedure that is used to process sequencing data generated from Illumina’s MiSeq platform using paired end reads.^1^ Barcodes and primers were removed first. Demultiplexing processes were conducted according to the following parameters: sequence length remains between 350 and 400 bp; number of ambiguous bases is 0; and maximum homopolymer length is 6. Quality-checked sequences were aligned against the aligned SILVA 128 database (eukaryotes only; Quast et al., 2013).^2^ The bulk of sequences that started at position 13,876 and ended at position 22,449 were extracted. These alignments were then trimmed using “vertical = T” and “trump =.” options to ensure that reads with the same primer set were aligned to the same exact region. A further screening step (pre.cluster) was applied to decrease sequencing noise by clustering reads that differ by 4 bp (diffs = 4). Chimeras were detected on individual samples using the UCHIME algorithm (Edgar et al., 2011) in a *de novo* setup and subsequently removed. For each sample, clean reads were dereplicated.

An operational taxonomic unit (OTU) table was constructed by clustering high-quality reads at a 3% genetic distance, based on the furthest neighbor cluster method (Logares et al., 2014). OTUs were then taxonomically classified using the RDP Classifier with a naïve Bayesian approach against a Protist Ribosomal Reference (PR^2^) database (Guillou et al., 2013) at a 80% confidence level. Distant OTUs with an *e*-value >10^−100^ (below ~80% similarity) were considered as “unknown” and were removed. Taxa that are not affiliated with protists (e.g., Bacteria, Archaea, Nucleomorphs, and Metazoa) were removed from the dataset before downstream analysis to avoid distortion of the relative abundance of DNA sequences of microbial eukaryotes. In this study, microeukaryotes are generally referring to pico-nanosized protists, but acknowledge that there may be some OTUs of microsized protists in our datasets (e.g., ciliates, dinoflagellates, and Radiolarians) that could derive from cell debris or extracellular DNA from these larger cells (Not et al., 2009; Kimoto et al., 2011).

In the final dataset, after filtering all unwanted sequences, only 188,003 reads remained with a mean of 3,133 sequences recovered per sample (ranging from 1,172 to 6,565). Normalization procedure under sub.sample command was conducted to enable comparison between samples in different sequencing coverage depths, based on the lowest sequence count (1,172 sequences) from the bottom layer of station C8 collected in October (10C8B). Finally, an OTU table, generated with the make.shared command in MOTHUR, was used to study the structure of microbial eukaryote communities in the 60 samples. Singletons, doubletons (i.e., OTUs with only one or two sequences), and OTUs present in a single sample were discarded from the OTU table. This was based on the assumption that they were likely sequencing errors or originated from sediment samples that were occasionally collected in a previous work (Logares et al., 2014).

### Definition and Classification of Microbial Taxa

In addition to the analysis of the whole communities, separate datasets of abundant and rare taxa were also identified to compare the differences between two sub-communities, relating to their richness and abundance, patterns of community structure, and biogeographic processes. Unlike previous studies that only considered abundant and rare taxa with varied thresholds depending on the cutoff level of relative abundance at local scale (usually at 1% for abundant OTUs, Galand et al., 2009; Logares et al., 2014 and at 0.1%, Dai et al., 2016 or 0.01%, Galand et al., 2009; Logares et al., 2014 for rare OTUs), the present study focused on intermediate taxa (i.e., relative abundance between 0.1 and 1%) and on oscillating taxa groups (i.e., rare and abundant under different conditions; Shade et al., 2014; Xue et al., 2018) considering the relative abundance variation of the same OTU across all samples (Dai et al., 2016). Therefore, as previously described (Dai et al., 2016; Chen et al., 2017; Xue et al., 2018), six categories were classified based on their range of relative abundance in this study: abundant taxa (AT) with a relative abundance ≥1% in all samples; conditionally abundant taxa (CAT) with a relative abundance exceeding 0.1% in all samples and ≥1% in some samples but never <0.1%; moderate taxa (MT) with a relative abundance ranging between 0.1 and 1% in all samples; conditionally rare and abundant taxa (CRAT) with a relative abundance ranging from rare (≤0.1%) to abundant (≥1%); conditionally rare taxa (CRT) with a relative abundance below 1% in all samples and ≤0.1% in some samples; and rare taxa (RT) with a relative abundance ≤0.1% in all samples.

### Statistical Analyses on PCCs’ Diversity and Biogeographic Processes

#### Diversity Analysis and Community Compositions

Rarefaction curves, alpha-diversity indexes, and species accumulation curves (SAC) were calculated in Vegan 2.5.5 package (Dixon, 2003) in R (v.3.6.1). Spatiotemporal effects on alpha-diversity were examined *via* two-way ANOVA (Xue et al., 2018) and non-parametric test (Kruskal-Wallis ANOVA) using SPSS 22.0 (IBM, Armonk, NY, United States). Spearman’s rank correlations were conducted to test the abundance-occupancy relationships (Mo et al., 2018). A heatmap showing relative abundance of protist lineages at lower taxonomic level were generated in the pheatmap package in R. Analyses of similarities (ANOSIM) were conducted in MOTHUR to assess the difference in PCCs between groups of samples based on Bray-Curtis similarity matrixes and the unweighted Unifrac metric, which were compared to verify the robustness of dissimilarity among communities. Microeukaryotic data were Hellinger-transformed before analyses to provide unbiased estimates (Legendre and Gallagher, 2001). Community dissimilarity was then visualized in non-metric multidimensional scaling (NMDS) based on the Bray-Curtis distance, which was assumed to be more suitable for the communities due to its capability to identify local differences among samples.

#### Evaluating the Effects of Environmental and Spatial Factors on Community Assembly

Before multiple statistical analyses, all environmental factors were square-root transformed to decrease non-normality and heteroscedasticity. The principal component analyses (PCA) were first conducted using Vegan package to test the explanation of all environmental variables on the variation of the samples. The potential relationships between environmental factors and PCCs were then established through Procrustes analysis (Peres-Neto and Jackson, 2001), that is, by adjusting the PCoA coordinates of PCCs to match the PCA coordinates of the environmental factors. The sum of squared deviations between the point coordinates (*M*^2^) was used to evaluate the community-environment correlation. The smaller the *M*^2^ was, the stronger the correlation was. Significance test of *M*^2^ was achieved by PROTEST (PROcrustean randomization test) in Vegan.

Correlation analyses between the environmental parameters and the abundance and diversity of the entire protist community, and between the environmental factors and 11 abundant protist lineages (percentage of reads >1% in all 60 samples) were tested using Spearman rank correlation coefficient in the Vegan package in R. A total of 37 protist lineages were included in the redundancy analysis (RDA) to further test and visualize the relationships between environmental variables and community compositions.

To help visualize the patterns of dispersal limitation and environmental filtering on PCCs, distance-decay curves were plotted by linear regression of the Bray-Curtis similarities of PCCs against the pair-wise geographical distances matrix based on longitudinal and latitudinal coordinates of each sampling site (Gong et al., 2015) and the Euclidean distances of the environmental factors among samples. A set of spatial factors was generated using the approach of the principal coordinates of neighbor matrices (PCNMs) based on the longitude and latitude coordinates of the sampled locations, using the R package PCNM (v.2.1-2; Legendre and Gauthier, 2014). Mantel tests were then conducted to examine the influences of environmental filtering and spatial factors on the PCCs’ biogeographic processes in Vegan package.

A neutral community model was used to assess the potential importance of neutral processes on the PCCs’ assembly by fitting the relationship between OTU occurrence frequency and their relative abundance (Sloan et al., 2006). In this model, *R*^2^ indicates the fitness to the neutral model and *Nm* values the metacommunity size (*N*) times immigration rate (*m*), indicating the dispersal between sub-communities (Sloan et al., 2006).

At last, to figure out the biotic interactions among three sub-communities and different protist lineages in the whole community, co-occurrence networks were constructed using the “WGCNA” R package based on Spearman correlation (Langfelder and Horvath, 2008, 2012; Pei et al., 2017). Values of *p* were adjusted by “*q*-values” using a modified version of false discovery rate test (Storey, 2003). Only the strongly significant pairwise correlations (|*r*| > 0.6, *p* < 0.05) among OTUs were kept for network construction. The networks were then visualized with the Gephi 0.9.2 interactive platform.^3^ The nodes and the edges in the network represent OTUs and the correlations between pairs of OTUs, respectively. Several indices of topological properties were computed in the “igraph” R package to evaluate the structure of the network (Deng et al., 2012).

## Results

### General Spatiotemporal Patterns of Abundance, Richness, and Diversity of Protist Communities

All environmental factors (including abiotic and biotic factors) differed significantly across time and space (one-way ANOVA, *p* < 0.05, data not shown). When pooling all environmental factors together in PCA plots, obvious seasonal difference was discovered in coastal area (Figure 1B). Samples from summer clustered clearly per habitat (shelf vs. coastal), whereas samples from spring and autumn did not show clear separations between habitats. The first two axes explained about 51.0% of the variance.

**Figure 2:**
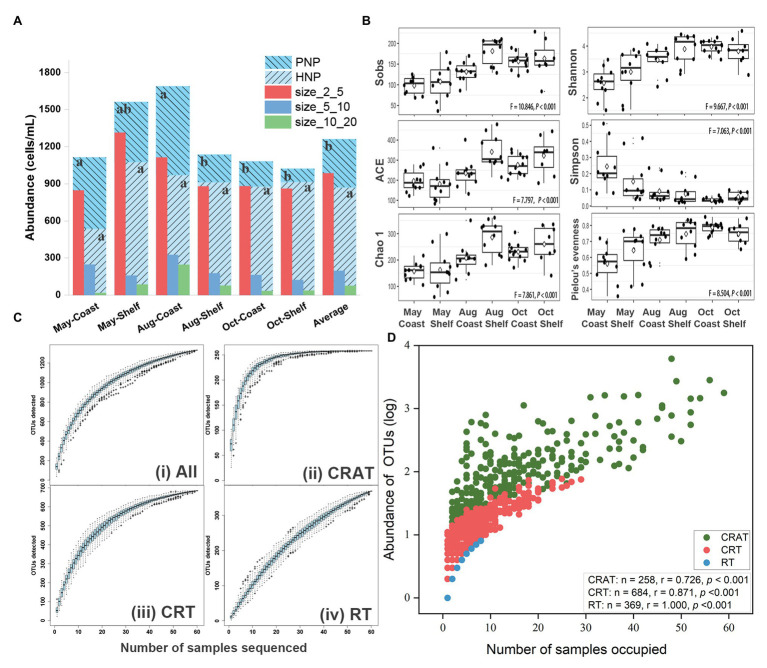
Abundance, diversity, and abundance-occupancy relationships of protist community. **(A)** Cell abundance, trophic structure, and size structure of nano-sized protist communities across time and space. Different letters denote significant differences among groups. **(B)** Boxplots displayed the temporal and spatial distribution of alpha-diversity index of protist communities. Non-parametric test (Kruskal-Wallis ANOVA) was used to compare the differences among six groups and all values of *p* were lower than 0.001. The hollow diamonds represent average values of individual index in each group. **(C)** Species accumulation curves (SAC) of protist taxa [i. all OTUs; ii. conditionally rare and abundant taxa (CRAT); iii. conditionally rare taxa (CRT); and iv. rare taxa (RT)]. Boxplots in SAC showed the range of OTU richness for each number of samples sequenced. **(D)** Abundance-occupancy relationships between OTU richness and number of sites occupied. Spearman’s rank correlations were used to evaluate the relationships between abundance of OTUs (log-transformed) from three different microbial subcommunities and the number of sites occupied (*n* is the number of OTUs).

For the trophic and size structures of the nano-sized protist community, in general, HNP was more abundant than PNP in most samples (Figure 2A and Supplementary Table S1). The abundance of NP was highest in the size class of 2–5 μm and the ratio between the three size categories was about 2–5 μm:5–10 μm:10–20 μm = 13:3:1 (Figure 2A). As a general trend, the richness and diversity indexes increased significantly (*p* < 0.001; Figure 2B) from May to October and from coastal area to shelf area. However, both the richness and diversity of surface and bottom layers did not differ significantly (Supplementary Figure S2).

After subsampling to 1,172 sequences per sample, 70,320 sequences (1,332 OTUs) were retained (Supplementary Table S2). The slightly increasing trends of the rarefaction curves (Supplementary Figure S1) and the SAC for All OTUs (Figure 2Ci) indicated that the majority of protist taxa were well recovered. The three sub-communities (AT, CAT, and MT) were not found based on the thresholds in this study and have not been considered in the downstream analyses (Supplementary Table S2). RT sub-community constituted only 1% of the total sequence number with high OTU richness (369 OTUs), whereas the abundance of CRAT constituted nearly 90% of the total sequence number with low richness (258 OTUs). The SAC of CRAT, CRT, and RT taxa showed different trends with a level-off line in CRAT and largely increasing in RT and CRT taxa. This implied nearly complete detection of dominant species, whereas much more rare taxa were not discovered (Figure 2Cii,Ciii,Civ). Two-way ANOVA indicated significant seasonality and habitat effects on the richness and diversity of the whole community (Table 1) and three sub-communities (Supplementary Table S3).

**Table 1:** Two-way ANOVA showing the effects of seasonality and/or habitats (coast and shelf area) on the α-diversity estimators of operational taxonomic units (OTUs; 97% similarity cut-off) of the whole communities.

Diversity estimators	Seasons		Habitats		Seasons × Habitats	
	*F*	*p*	*F*	*p*	*F*	*p*
Sobs	**21.692**	**0.000**	**8.152**	**0.006**	2.995	0.060
ACE	**12.563**	**0.000**	**5.942**	**0.019**	**3.261**	**0.047**
Chao1	**14.731**	**0.000**	**6.358**	**0.015**	2.066	0.138
Shannon	**19.792**	**0.000**	2.322	0.134	1.653	0.202
Simpson	**13.613**	**0.000**	1.513	0.225	1.787	0.179
Pielou’s	**16.509**	**0.000**	1.287	0.262	2.053	0.140

Significant values of *p* (<0.05) are highlighted in bold. *F*, F-statistic; *p*, values of *p*.

**Figure 3:**
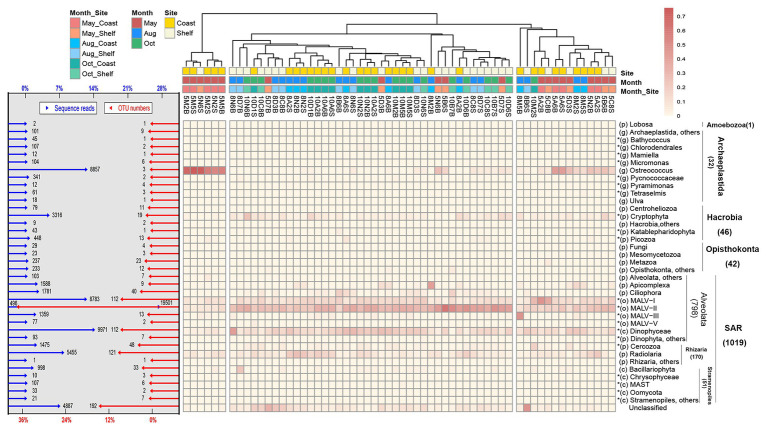
The distribution and relative abundance of different taxonomic lineages in the entire protist community in 60 samples. The OTUs were defined at 97% sequence similarity threshold. Heatmap showed the relative abundance of the 37 lineages which were classified into seven supergroups within each sample; each of the lineages that contained more than 10 sequence reads within the entire dataset was individually presented, whereas the rare lineages (fewer than 10 sequence reads) and the undetermined lineages (sequence similarity >80%) merged as “others.” Sequence reads are “unclassified” when sequence similarity to a reference sequence is <80%. The OTU numbers are given in parentheses for each supergroup. The rank classification of each lineage is listed before with lowercase in bracket (“p” for phylum, “c” for class, “o” for order, and “g” for genus). An asterisk in front of the taxon indicates the lineages belonging to flagellates. Bar graphs in the left showed the relative abundance of sequence reads (species abundance) and OTU numbers (species richness) in the 37 branching lineages within the entire dataset (metacommunity including 60 local communities). Dendrogram on the top showed the clustering for 60 samples based on Bray-Curtis similarity. The color codes at the branch end were based on months, habitats, and both of them.

With regard to the whole community, a total of 192 OTUs with 4,887 reads (14.4% of richness and 6.9% of abundance) could not be classified into specific taxa based on >80% sequence similarity and consequently being identified as unclassified (Figure 3). In fact, more unclassified OTUs were found in CRT (15.67%) and RT sub-communities (15.99%) compared with the CRAT sub-community (9.30%; Supplementary Figure S3). The remaining assigned 1,140 OTUs were classified into 37 deep-branching lineages among seven supergroups (Alveolata, Amoebozoa, Archaeplastida, Hacrobia, Opisthokonta, Rhizaria, and Stramenopiles; see Figure 3 for the names of all 37 lineages). The lineages were classified mainly based on Adl et al. (2012). The relative richness (numbers of OTUs) and abundance (numbers of sequences) of various taxonomic lineages were differently distributed over time and space (Figure 3). *Ostreococcus* was dominant in May, whereas Cryptophyta had higher abundance in October. However, MALV-II, MALV-I, Dinophyceae, and Radiolaria were the four most diverse and abundant groups in the total community and three sub-communities in most samples (Figure 3 and Supplementary Figure S3).

### Biogeographic Patterns of Protist Community Structure

**Figure 4:**
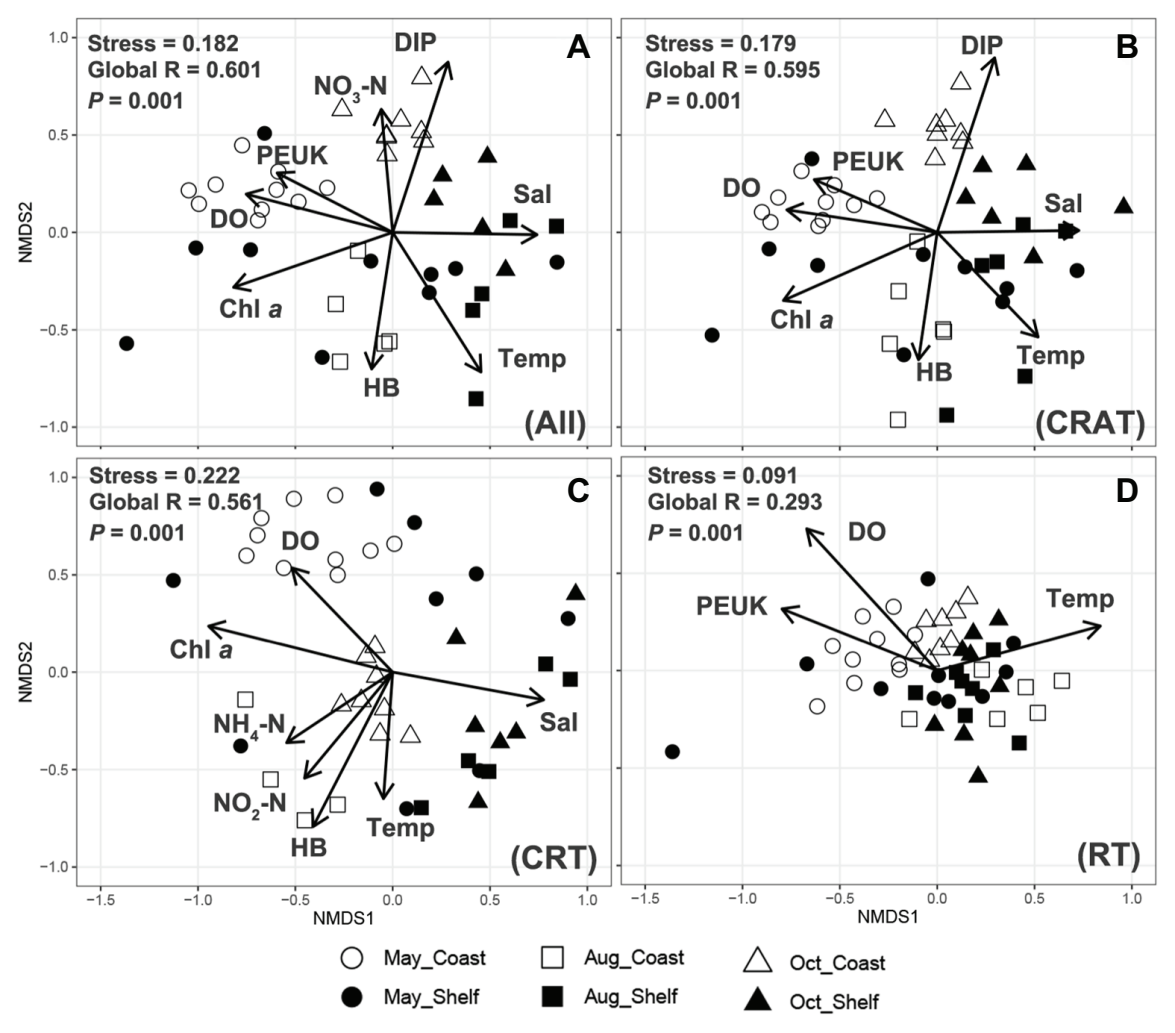
Non-metric multidimensional scaling analysis showing the biogeographic distributing patterns of protist communities in the studied area and the significant environmental factors (*p* < 0.01) that influence the communities. The same clustering of samples into six groups across three seasonally representative months and two habitats is shared by the entire community (**A**. All) and three sub-communities (**B**. CRAT, **C**. CRT, and **D**. RT), based on Bray-Curtis dissimilarity between samples.

No individual OTU was identified in all 60 samples. The number of OTUs decreased sharply with increasing shared samples (Supplementary Figure S4C). Only 39 OTUs (2.93%) were found in more than 30 samples, and more than half of the OTUs (728, 54.65%) were found in less than three samples (Supplementary Figure S4C). This confirmed that only a small number of species can be hyper-abundant (Keeling and del Campo, 2017) with wide and ubiquitous distribution. Abundance-occupancy relationships between the abundance of individual OTUs (log-transformed) of three sub-communities and the number of occupied sites correlated significantly positively (Spearman’s rank correlations, *r* > 0, *p* < 0.001; Figure 2D). CRAT taxa occupied 2–59 samples whereas CRT taxa occupied no more than half of the samples (maximum 29 samples), whereas RT taxa only occupied a maximum of eight samples (Figure 2D), indicating ubiquitous or restricted distribution of individual OTUs. The results were confirmed by Venn plots that the percentages of shared OTUs decreases in the order of CRAT, CRT, and RT (Supplementary Figure S5). At higher taxonomic level, spatiotemporal biogeographical patterns were found for the entire community (Figure 3). This was also true at the OTU level where samples, collected in two habitats (coast and shelf) from a given month, clustered (NMDS; Figure 4A). This biogeographic pattern was similar among CRAT, CRT, and RT sub-communities (Figures 4B–D). However, seasonal separation was not as distinguishable for samples from the shelf area as for samples from the coastal area (Figures 4A–D). This was verified by hypothesis testing with the global R being closer to 0 among different seasons in shelf area no matter which distance was used (ANOSIM; Table 2 and Supplementary Table S4). Furthermore, when considering the difference of PCCs between surface and bottom layers, the global R were relatively lower compared with seasonal or habitat differences, especially in coastal area and spring and autumn (*p* > 0.05; Table 2 and Supplementary Table S4). This was mainly due to the shallow depth of coastal areas and well-mixed water in spring and autumn. In contrast, high temperature of the surface water and large freshwater runoffs in summer may cause stratification phenomenon and reduce vertical water exchange (Wang et al., 2012), thus resulting in different PCCs above/beneath the thermocline (*p* < 0.05; Table 2 and Supplementary Table S4).

**Table 2:** Analysis of similarities (ANOSIM) results of different comparisons based on months (May, August, and October), layers (B for bottom and S for surface), and habitats (coast and shelf) among the whole protist communities based on unweighed Unifrac distance and Bray-Curtis distance.

Distance between samples		Unweighted Unifrac		Bray-Curtis	
Grouping by	Comparisons	*R*	*p*	*R*	*p*
Month	Aug vs. May	0.457	<0.001^*^	0.492	<0.001^*^
	Aug vs. Oct	0.340	<0.001^*^	0.316	<0.001^*^
	May vs. Oct	0.515	<0.001^*^	0.432	<0.001^*^
	Aug_B vs. May_B	0.418	<0.001^*^	0.430	0.001^*^
	**Aug_B vs. Oct_B**	**0.210**	**0.011**	**0.180**	**0.028**
	May_B vs. Oct_B	0.358	<0.001^*^	0.398	<0.001^*^
	Aug_S vs. May_S	0.523	<0.001^*^	0.632	<0.001^*^
	Aug_S vs. Oct_S	0.548	<0.001^*^	0.599	<0.001^*^
	May_S vs. Oct_S	0.731	<0.001^*^	0.539	<0.001^*^
	Aug_Coast vs. May_Coast	0.844	<0.001^*^	0.946	<0.001^*^
	Aug_Coast vs. Oct_Coast	0.814	<0.001^*^	0.920	<0.001^*^
	May_Coast vs. Oct_Coast	0.982	<0.001^*^	0.944	<0.001^*^
	Aug_Shelf vs. May_Shelf	0.245	0.003^*^	0.297	0.001^*^
	**Aug_Shelf vs. Oct_Shelf**	**0.074**	**0.144**	**0.130**	**0.065**
	May_Shelf vs. Oct_Shelf	0.274	0.004^*^	0.347	0.001^*^
Layer	B vs. S	0.127	<0.001^*^	0.120	<0.001^*^
	Aug_B vs. Aug_S	0.249	0.003^*^	0.230	0.006^*^
	**May_B vs. May_S**	**0.058**	**0.131**	**0.141**	**0.022**
	**Oct_B vs. Oct_S**	**0.053**	**0.134**	**0.051**	**0.169**
	**Coast_B vs. Coast_S**	**0.001**	**0.370**	**−0.020**	**0.575**
	Shelf_B vs. Shelf_S	0.302	<0.001^*^	0.396	<0.001^*^
Habitats	Coast vs. shelf	0.232	<0.001^*^	0.304	<0.001^*^
	Aug_Coast vs. Aug_Shelf	0.361	<0.001^*^	0.401	<0.001^*^
	May_Coast vs. May_Shelf	0.321	<0.001^*^	0.382	<0.001^*^
	Oct_Coast vs. Oct_Shelf	0.599	<0.001^*^	0.694	<0.001^*^
	Coast_B vs. Shelf_B	0.333	<0.001^*^	0.428	<0.001^*^
	Coast_S vs. Shelf_S	0.188	0.003^*^	0.284	<0.001^*^

Bold fonts represent insignificant difference with values of *p* higher than 0.01. ^*^Correlation is significant at the 0.01 level.

Spearman’s rank correlations comparing Bray-Curtis community similarity vs. geographic distances and the Euclidean distances of the environmental factors among samples indicated weak (*r* < 0.3) but significant (*p* < 0.01) negative correlations for the entire community, as well as for CRAT, CRT, and RT sub-communities (Supplementary Figure S6). These exhibited similar distance-decay patterns in the similarities of both abundant and rare sub-communities, with increasing geographical distances and environmental dissimilarities.

### Effects of Environmental and Spatial Factors on Protist Communities

In general, the abundance (represented by NP) and diversity (represented by Chao 1, Shannon, and Pielou’s evenness) of PCCs have opposite correlations with different environmental factors (Supplementary Table S5). The abundance of NP had a significantly negative relationship with salinity (*r* = −0.492, *p* < 0.01) and a positive relationship with DO (*r* = 0.329, *p* < 0.01) and Chl *a* (*r* = 0.412, *p* < 0.01), whereas the alpha diversity displayed opposite trends. Furthermore, directly negative correlations were also found between diversity and abundance (Supplementary Table S5). This result indicated that environmental factors may indirectly affect the diversity of the PCCs by affecting their abundance (Caron and Hutchins, 2012).

The redundancy analysis plot showed that environmental factors were related to the distribution of different protist taxa, thus leading to different spatiotemporal distributions of the PCCs (Figure 1C). Compared with other dominant taxa, *Ostreococcus* showed an opposite pattern of distribution and was extremely dominant in May, corresponding to the lower temperature, salinity, and nutrients and the higher DO and Chl *a* in May (Figures 1C,D). Similar results were observed in other studies on the distribution of *Ostreococcus* (Countway and Caron, 2006; Wu et al., 2017). MALV-III, Radiolaria, Dinophyceae, and Apicomplexa tended to be abundant in coastal area in August, responding to high temperature, nutrition, Chl *a*, and abundance of food sources such as heterotrophic bacteria (HB) and *Synechococcus* (Figure 1C). Other lineages such as MALV-II, Cryptophyta, Ciliophora, and Bacillariophyta displayed dominance in October, responding to high phosphorus and low Chl *a* (Figures 1C,D). Furthermore, the relationships between significant environmental factors (*p* < 0.01) and PCCs at the OTU level (Figure 4) indicated that both of the abiotic drivers and biotic interactions were related to the variation of PCCs. Eight environmental factors (i.e., DIP, Chl *a*, temperature, DO, salinity, PEUK, HB, and nitrate) were significantly related with variations in the entire community (*p* < 0.01).

**Figure 5:**
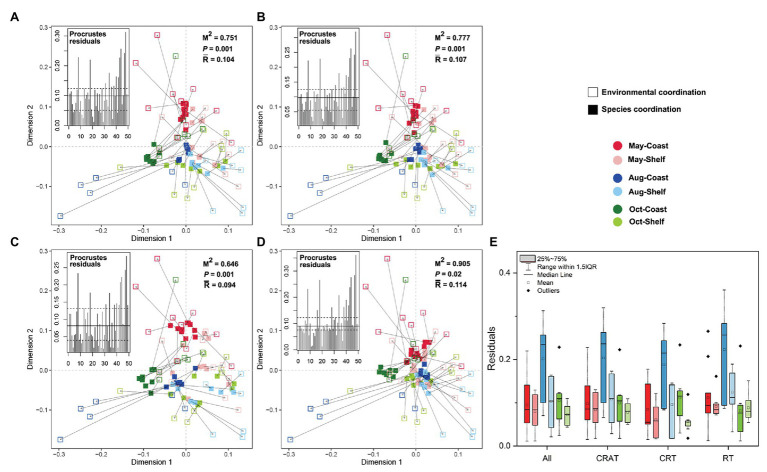
Procrustes analyses of the correlations between environmental PCA coordination and community compositions’ PCoA coordination (based on Bray-Curtis distances) of each taxa dataset (**A**. All; **B**. CRAT; **C**. CRT; and **D**. RT; *M*^2^, the Procrustes sum of squares; *p*, the significance under 999 permutations test; and *R*, the mean value of Procrustes residuals). The Procrustes residuals of each sample are shown at the left top and of six sampling groups are compared in the boxplot **(E)**.

The results of the Procrustes analysis showed good consistency of the potential relationship between the PCCs and environmental variations with significant correlations (PROTEST, *p* < 0.05; Figures 5A–D), especially in CRT sub-community (*M*^2^ = 0.646, Residuals = 0.094). Moreover, the Procrustes residuals displayed significantly higher values in coastal areas (one-way ANOVA, *p* < 0.05), with the highest in August-coast (Figure 5E), implying increasing association between the PCCs and environment from coastal area to shelf area.

**Figure 6:**
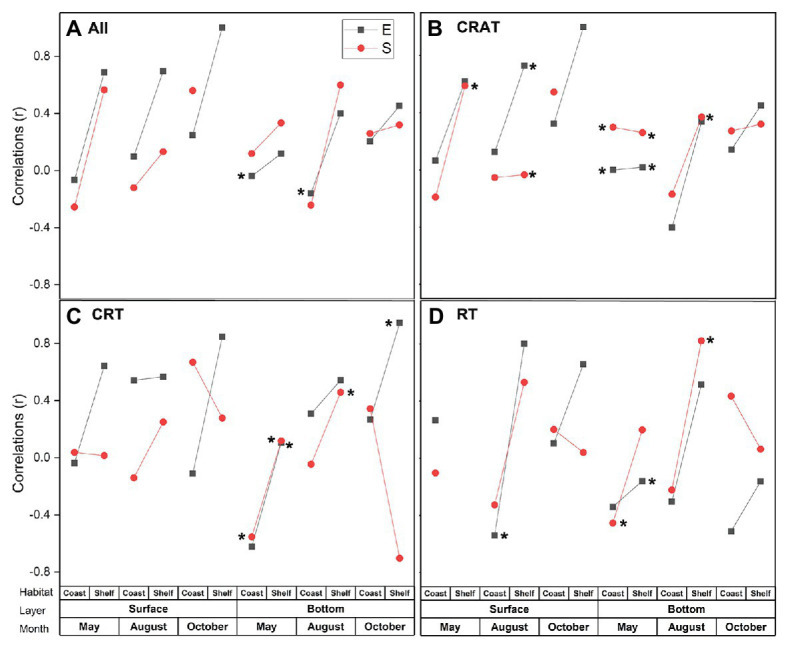
Mantel tests for the correlations among environmental distance (E), spatial distance (S), and protist communities’ distance (**A**. All; **B**. CRAT; **C**. CRT; and **D**. RT) in surface and bottom layers in coastal and shelf areas in each season using Spearman’s rank coefficients. Asterisks besides the dot indicate significant correlations (*p* < 0.1).

The Mantel tests showed significant correlation between environmental and temporal factors (*p* < 0.05; Supplementary Table S6), indicating strong seasonality of environmental variables in this study. However, no significant correlation was found between environmental variables and geographic distance (Supplementary Table S6 and Supplementary Figure S7). Therefore, simple mantel tests for surface and bottom layers in coastal and shelf areas in different seasons were conducted separately to assess the effects of environmental and spatial factors on PCCs (Figure 6). In general, environmental factors exerted stronger relationships with PCCs than spatial factors in most cases. The correlations between PCCs and both of the environmental and spatial factors increased from coastal area to shelf area (Figure 6A). Similar patterns of explanations were displayed in CRAT, CRT, and RT communities in most cases (Figures 6B–D). Meanwhile, the correlations of both the environmental and spatial factors displayed increasing trend from May to October and from bottom layer to surface layer generally (Supplementary Figure S8).

**Figure 7:**
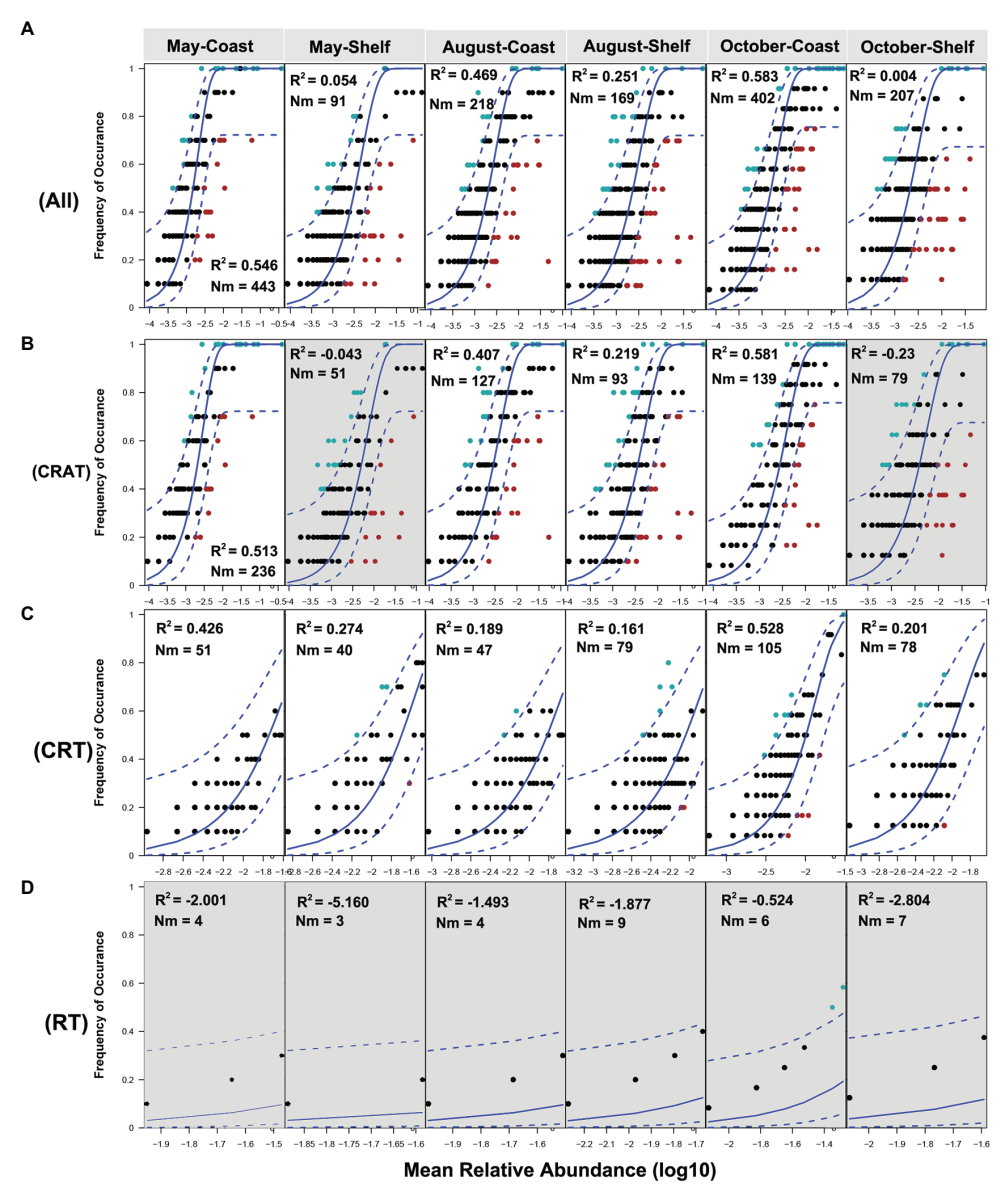
Fit of the neutral model of community assembly. The predicted frequencies of OTUs from the set of the whole community (**A**. All), CRAT **(B)**, CRT **(C)**, and RT **(D)** sub-communities in coastal and shelf areas in 3 months are displayed, respectively. The solid blue lines indicate the best fit to the NM and dashed blue lines represent 95% CIs around the model prediction. OTUs that occur more or less frequently than predicted by the NM are shown in different colors. *Nm* indicates the size of the metacommunity (*N*) times immigration rate (*m*), *R*^2^ indicates the fit to the *Nm*. Note that the datasets with negative *R*^2^ value indicate no fit to the neutral model, of which the backgrounds are in gray.

The neutral model explained a larger fraction of the variation (*R*^2^) in occurrence frequency of the whole community in coastal area than in shelf area (Figure 7). The same trends were also discovered in CRAT and CRT sub-communities (Figures 7B,C). Meanwhile, the fitness and the *Nm* values of CRAT sub-communities were larger than CRT in most cases. The RT sub-community exhibited no fit to the neutral curve (Figure 7D, *R*^2^ < 0; negative *R*^2^ values can occur when there is no fit to the model), indicating the weakest stochastic neutral processes in RT sub-communities.

### Protists’ Co-occurrence Networks

**Figure 8:**
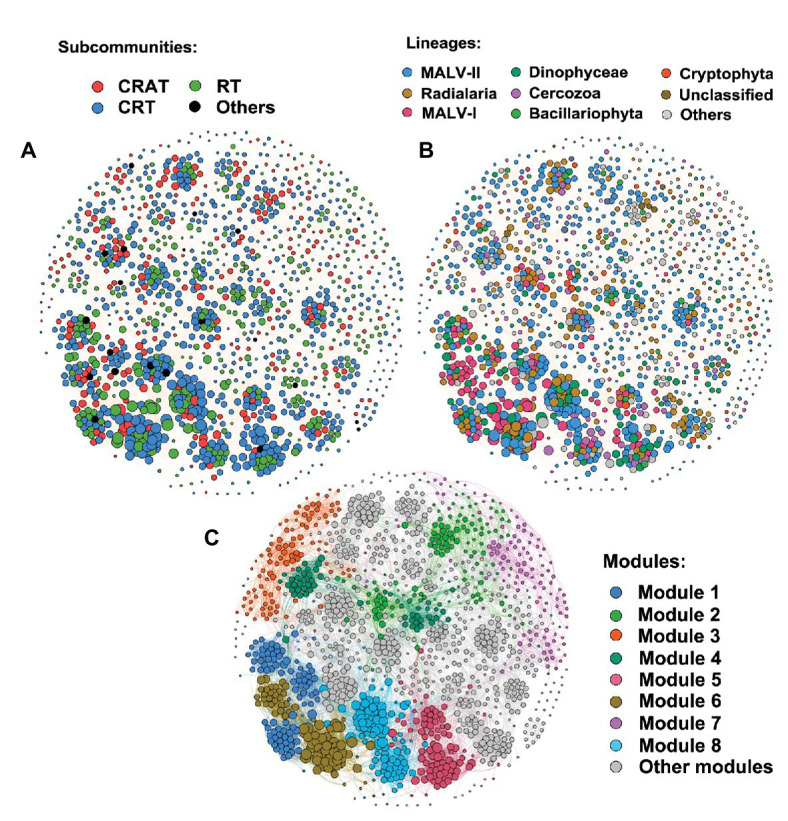
Co-occurrence networks among OTUs of protist community. The nodes were colored according to different categories of sub-communities **(A)**, taxonomic lineages **(B)**, and modules **(C)**. Connections between two individual nodes stand for strong (Spearman’s *r* > 0.6 or *r* < −0.6) and significant (*p* < 0.05) correlation. The size of each node is proportional to the number of connections (i.e., degree).

The present study explored the co-occurrence network that formed by strongly significant pairwise correlations (|*r*| > 0.6, *p* < 0.05) among taxa, to search for how biotic interactions could be important to structure the PCCs in different conditions (Figure 8 and Supplementary Figure S9). OTUs belonging to CRAT, CRT, and RT sub-communities formed strong intra‐ and inter-associations with themselves and with each other. In general, the whole community had more than eight sub-modules (i.e., taxa with a high co-occurrence in similar niche), which was composed of substantial rare taxa and a few abundant taxa in multiple taxonomic lineages (Figure 8). It appeared that the networks from coastal area held more sub-modules with lower connectivity and average degree than shelf area (Table 3 and Supplementary Figure S9). This indicated complex community structure and obvious “small world” properties in coastal area due to high degree of environmental heterogeneity (Deng et al., 2012). We also observed higher contributions and degree of connections (i.e., the size of each node) of CRT and RT sub-communities compared with CRAT in the biotic associations within modules (Figure 8), indicating the importance of rare biosphere which contributes a large part in the whole community.

**Table 3:** Properties of the co-occurrence networks of protist community compositions (PCCs) in the East China Sea (ECS).

Network index	May-Coast	May-Shelf	Aug-Coast	Aug-Shelf	Oct-Coast	Oct-Shelf
Number of vertices	322	515	477	797	523	608
Number of edges	3,142	13,232	7,826	22,360	6,722	15,038
Average degree	19.516	51.386	32.813	56.11	25.706	49.467
Betweenness centralization	0.362	0.098	0.100	0.093	0.113	0.163
Average clustering coefficient	0.874	0.919	0.878	0.907	0.824	0.898
Diameter	27	11	12	13	12	15
Average path length	8.275	4.323	5.040	4.811	5.25	5.789
Density/connectivity	0.061	0.100	0.069	0.070	0.049	0.081
Degree centralization	0.108	0.102	0.059	0.045	0.080	0.067
Modularity	0.769	0.770	0.836	0.871	0.800	0.833
Number of sub-modules	39	13	24	21	38	15

## Discussion

### Dominance of HNP Over PNP Under Microscopic Observation

In this study, the dominance of HNP over PNP was found under the microscopic observation in most sampling sites. This goes against most of the previous studies conducted in coastal waters which showed higher abundance of pigmented nanoflagellates than heterotrophic nanoflagellates (Granda and Anadon Alvarez, 2008; Jürgens and Massana, 2008; Tsai et al., 2013). We can think of two non-exclusive explanations for our findings. First, all cells below 2 μm (i.e., pico-sized) were excluded in the counting procedure as we only focused on the abundance of nano-sized protists, which may decrease a large fraction of the abundance of pigmented protist. It is well known that a large part of the pigmented cells of protists are within the small size fraction (Vaulot et al., 2008). Second, increased exposure to UV radiation leads to higher oxidative stress in the surface water, explaining the fact that less pigmented cells are found in this water than potentially in the deepest waters (in particular in the DCM, i.e., deep chlorophyll maximum; Nunez et al., 2006). Furthermore, previous studies have found that exclusively heterotrophic protists are more common in natural ecosystems and consist of a larger number of taxa compared with pigmented protists (Jürgens and Massana, 2008). Similar results were further retrieved in our sequencing dataset, where we found that the total relative abundance of lineages belonging to HNPs (e.g., MALV-I, MALV-II, ciliates, radiolarians, and Cercozoan) was higher than that of PNPs (e.g., Dinoflagellate, Diatom, and Cryptophyta; Figure 3). This indicated good agreement and complementarity of molecular and microscopic methods in estimating the relative abundance of species composition, although the species abundance (number of sequences) obtained by molecular method differs from the absolute cell numbers observed by microscope by several orders of magnitude (Lejzerowicz et al., 2015; Santoferrara et al., 2016; Liu et al., 2017). Overall, it is possible that the observed abundance of HNP exceeded PNP in some cases. This result provides a pertinent evidence to combine the molecular method with microscopic observation in the study of protist community structure.

### Different Diversity but Similar Biogeographic Patterns Among CRAT, CRT, and RT Sub-communities

In this study, we defined six categories of taxa based on their abundance across all samples, with the consideration of intermediated taxa and oscillating taxa. However, it was interesting to find that no species fit the AT (always abundant), MT (always moderately abundant and never rare), or CAT (never rare) categories. First, there is always an effect of arbitrariness when defining a threshold for rarity because “rarity” is considered to be a continuous variable in the ecological studies (Chen et al., 2017). Thus, it is just a function of how the cutoff point of “rarity” is defined. However, it is still noteworthy that there were no universally abundant taxa and “conditionally” or “moderately” abundant but never rare taxa that existed in the multidimensionally spatiotemporal scales of this research. In other words, the community composition is oscillating across time and space due to the dynamic changes in environmental conditions. Thus, “conditionally rare and abundant” taxa (CRAT) are prevalent which could adjust their richness in response to environmental conditions and stochastic processes. Finally, according to some studies (Nino-Garcia et al., 2016a, 2017), maintaining the state of rarity may be a survival strategy for some always rare species (RT) and conditionally rare species (CRT) in some cases, whereas it is also possible to gain superiority under other spatiotemporal conditions that were not covered in this study.

The diversity, including the richness (numbers of OTUs) and abundance (numbers of reads) of three identified sub-communities (CRAT, CRT, and RT), were clearly different, as were the taxonomic compositions. However, MALV-II, MALV-I, Dinophyceae, and Radiolaria were the four most diverse and abundant groups in all three sub-communities. This result is similar to a study of abundant and rare pico-eukaryotic sub-communities (Wu et al., 2016), which described dominant compositions of Dinophyta and Radiolaria in epipelagic waters in marginal seas. This may indicate not only dominant-and-active roles of Dinophyta and Radiolaria as abundant taxa (Xu et al., 2017) but also rare-but-buffering roles as rare taxa (Caron and Countway, 2009). Moreover, the dominant taxonomic groups may transfer and shift from coast to shelf, from surface to bottom, and through different seasons under varied environmental or hydrographical conditions. Which of these combinations applies depends on their relative dispersal capacity (habitat usage or resistance to dispersal limitation) and their response to environmental changes (habitat adaptation, which is also known as species sorting; Wang et al., 2015; Yeh et al., 2015; Marquardt et al., 2016). In summary, it is the intrinsic properties of the different species and ecological groups that play an important role in community assembly (Logares et al., 2015).

Recent studies have detected biogeographical patterns of abundant and rare microeukaryotic sub-communities in different aquatic and terrestrial systems or ecological processes. Examples are freshwaters (Li et al., 2017), estuarine (Lallias et al., 2015), coastal waters (Chen et al., 2017), marginal seas (Wu et al., 2016), deep oceans (Pernice et al., 2016), the Arctic Ocean (Monier et al., 2014), a reservoir cyanobacterial bloom (Xue et al., 2018), and intertidal sediment (Chen et al., 2017; Zhang W. J. et al., 2018). Similar biogeographic patterns were identified in abundant and rare taxa in many cases (Chen et al., 2017), implying that rare taxa are likely subject to similar ecological processes (i.e., environmental variables and stochastic processes) that control abundant taxa. However, other studies reported distinct biogeographic patterns between abundant and rare taxa (Wu et al., 2016; Xue et al., 2018; Zhang Y. M. et al., 2018). In this study, the biogeographic patterns of CRAT, CRT, and RT sub-communities were similar but not completely identical. They showed different biodiversity patterns among groups, conspicuous separation among three seasons and two habitats, similar distance-decay pattern with spatial distance – presumably due to dispersal limitation (Hanson et al., 2012), and similar weak but significant relationships with environmental variables. In general, the abundant sub-communities have stronger dispersal ability than rare sub-communities and are more likely to be affected by environmental factors. Correspondingly, the rare sub-communities are more likely to be restricted in limited samples by dispersal limitation due to low dispersal ability. Similar patterns were found in abundant and rare sub-communities of picoeukaryotic metacommunity dynamics in the epipelagic waters of marginal seas (Wu et al., 2016) that rare taxa appear to be present only in few samples, whereas abundant taxa are more likely to move from one site to another as the result of prevalent current transportation.

The exception occurred to CRT sub-communities in the shelf area in May and October, where the limitation of spatial factors decreased, thus leading to higher dispersal ability in CRT (Figures 6C, 7C). This could be explained by the disproportionate contribution of CRT to the whole community (Shade et al., 2014), with a high proportion of OTUs (51.35%) with relatively low relative abundance (10.68%). They are usually constrained by environment or other specific dispersal limitations due to low growth rate or other life strategies; however, they may display distinct biogeographic process in some periods and modify the whole community under certain conditions (Shade et al., 2014; Lynch and Neufeld, 2015; Shade and Gilbert, 2015; Nino-Garcia et al., 2017). The study of the persistence of spatial abundant or rare taxa over time suggested intrinsic properties of particular taxa in lakes both in spatial and temporal scales (Nino-Garcia et al., 2017).

Except CRT, CRAT sub-community also displayed oscillating distribution patterns in the studied space and time scales. Identified as conditionally abundant and rare taxa, OTUs that belong to CRAT displayed fluctuating distributions of abundance in 60 samples (Supplementary Figure S4A), indicating conditionally adaptive ability in the changing environments. This was also the reason why there was no abundant taxa (AT) that exists in all 60 samples. Specifically, CRAT taxa tended to be concentrated and became abundant (>1%) in one or two random samples, meanwhile, becoming intermediate (from 0.1 to 1%) or rare (<0.1%) in at least one fifth of the samples (Supplementary Figure S4B). Positive abundance-occupancy relationships in CRAT (Figure 2D) could be explained by their intrinsically high growth rates that are able to be tolerant to wide environmental ranges and have strong response to environmental variability (Nino-Garcia et al., 2016b, 2017), as well as by high dispersal ability or probability of CRAT taxa (Nino-Garcia et al., 2016b), which results in a widespread or ubiquitous distributions in the sampled field.

### Controlling Mechanisms of Environmental and Spatial Factors on Protist Communities’ Assemblages Across Time and Space

The underlying mechanisms of the PCCs’ assembly controlled by deterministic processes (e.g., environmental filtering) and stochastic processes (e.g., dispersal, drift) still remain unclear and complex, although many studies have attempted to address these issues (Bahram et al., 2016; Wu et al., 2018). Some studies reported a more important role of environmental filtering on microeukaryotic community assembly (Hanson et al., 2012; Lallias et al., 2015; Wu et al., 2018), whereas other studies suggested greater effect of stochastic processes (Monier et al., 2014; Bahram et al., 2016) or a combined effect (Mo et al., 2018; Xue et al., 2018; Zhang W. J. et al., 2018). The results presented in this study suggested that environmental factors (Mantel test, *r* = 0.306) contributed more in structuring the protist metacommunity than spatial factors (Mantel test, *r* = 0.254). This pattern was consistent with the generally observed situation that communities collected at intermediate scales (tens to thousands of kilometers) under heterogenetic environmental conditions tended to be affected more by environmental factors (Martiny et al., 2006; Hanson et al., 2012; Langenheder and Lindström, 2019).

Strong seasonality of the variation on environmental conditions (Mantel test *r* = 0.333, *p* < 0.01) and PCCs (Mantel test *r* = 0.132, *p* = 0.013) occurred in the coastal area. On one hand, the coastal areas of Changjiang plume are directly influenced by CDW carrying high nutrient and low salinity currents and leading to large annual change in water temperature as well (Figure 1B). Meanwhile, fishing or other anthropogenic activities are intense around the coastal area and thus have an impact on the ecological environment (Li and Daler, 2004). On the other hand, the strength and direction of CDW vary in different seasons due to seasonal hydrographical and climatological properties such as multiple water currents and wind stress (Lee and Chao, 2003), changing the environmental conditions of coastal area seasonally. This might be the reason for more distinct seasonal patterns of PCCs in coastal area than in shelf area. Similar patterns were found in bacterial metacommunity dynamics in the southern ECS, depending on seasonal hydrography (Yeh et al., 2015). Temperature, salinity, DO, Chl *a*, nutrient, HB, and PEUK showed significant effects on PCCs no matter at higher taxonomic level or OTU level. Temperature is considered to be a main factor that drives the seasonal variation in temperate coastal area of Changjiang plume and its adjacent waters (Lee and Chao, 2003). Salinity and nutrient play critical roles in shaping the PCCs in the estuarine-coastal area subjected to freshwater discharge (Liu et al., 2018; Xu et al., 2020). An annual cycle of hypoxia observed in the Changjiang plume were coupled with anthropogenic nutrient loading and coastal eutrophication (Zhu et al., 2011) and with changes in the region of stratification (Wang et al., 2012), which may induce the growth of protists due to their tolerance of low O_2_ levels (Caron and Hutchins, 2012). All the factors mentioned previously could have an impact on protist grazing on picoplankton (i.e., HB and PEUK; Choi et al., 2012) and result in “bottom-up” controls on the PCCs (Caron and Hutchins, 2012). Notably, the environmental factors explained a larger proportion of the community variation in our samples (78.52%, Figure 1C), compared with previous studies (Mo et al., 2018; Xu et al., 2020). However, more key factors (e.g., pH, irradiation, turbidity, and particulate organic matters) were not included and should be considered in the future study of the complex coastal area (Choi et al., 2012).

Procrustes analysis showed better consistency of the community-environment relationship in shelf area, further supported by stronger correlation coefficients computed in the Mantel tests. As stated previously, different protist lineages and sub-communities can respond differently to the environmental factors, thus leading to different spatiotemporal distributions of PCCs. However, dramatic seasonal variation of environmental conditions in coastal area could also weaken the responding of the biological communities because the environmental factors can have a direct or indirect impact on the community which is a combined effect from multiple environmental factors (Caron and Hutchins, 2012). As a result, it takes time for the biological communities to respond and adapt to the rapidly changing environment. In other words, the responding of the community may lag behind the environmental variation. In contrast, it seems more easily for the protist communities to live in the open shelf area due to the relatively constant and gentle environmental conditions among seasons (Mantel test *r* = −0.024, *p* = 0.614). Furthermore, we observed higher Shannon diversity of PCCs in shelf area than in coastal area. According to the studies, the higher diversity could improve stability of the community to some extent (Konopka, 2009; Beyter et al., 2016). In summary, good consistency of the potential relationship between the PCCs and environmental variations depends on how the dynamics in the protist community assembly matches with the changing environment conditions.

Except for the environmental factors, increasing importance of the spatial factors on PCCs from coastal to shelf area was also observed. It appears that multiple water masses from varied directions meet in the shelf area and the whole current system becomes relatively weak as stated previously, thus inducing dispersal limitation of the protist communities (Lee and Chao, 2003). In contrast, high connectivity in the coastal area due to strong current transportation could weaken the dispersal limitation and cause high dispersal ability (Xu et al., 2020). Furthermore, correlations between the environmental factors and community decreased considerably from the surface to the bottom layer, thus the spatial factors displayed relatively more important role in the bottom layer. This result was consistent with the studies on the picoeukaryotic community (Wu et al., 2016) and the bacterial communities (Zhang et al., 2014) in both surface and subsurface or deep waters of the marginal China sea. Patch connectivity (Yeh et al., 2015) indicates that communities in the bottom layer were more stable and restricted by dispersal limitation (Hanson et al., 2012) due to the high independency and poor connectivity of bottom waters, which barely driven by the surrounding water masses or wind dispersal compared with well-connected patches in the surface layer (Ichikawa and Beardsley, 2002). Therefore, the communities are more likely to be selected by environmental filters during dispersal processes in the surface layer (Gilbert et al., 2012; Wang et al., 2015).

The neutral model of community assembly was usually conducted to detect the explanative variation of stochastic processes on protist communities (Chen et al., 2017; Xu et al., 2020). According to the neutral theory, ecological drift (i.e., stochastic processes of birth, death, colonization, and extinction) and evolutionary drift (i.e., stochastic genetic change) could contribute to purely spatial effects and unexplained variation (Hanson et al., 2012). Larger fitness (*R*^2^) and *Nm* values in coastal area further confirmed the stronger dispersal ability and the dominant role of stochastic processes in coastal area. High explanation of neutral process to the variation in PCCs was also found along the south coastal area in China and ascribed to the high and random dispersal rate of microeukaryotes affected by Zhe-Min Coast Current (Xu et al., 2020). Moreover, the RT sub-community exhibited no fit to the neutral curve, implying strong dispersal limitation due to weak dispersal ability of the rare taxa, further indicating more complex community assembly process in the RT sub-community. This result was inconsistent with the Mantel test where spatial factors (representing dispersal limitation) displayed lower correlations with RT sub-communities. Such inconsistency may be attributed to more complex stochastically related processes that could be included and explained with neutral model (Sloan et al., 2006). However, the Mantel tests only consider the geographic distance of sampling sites as the spatial factor, while ignoring other drift processes.

In this study, both the local environmental conditions and dispersal processes have an impact on structuring PCCs in many cases; however, their effects can change through time. This may be the result of different hydrographical properties and physical dispersal strengths in different seasons that affect the environmental heterogeneity and connectivity of the communities (Lee and Chao, 2003; Yeh et al., 2015). In this case, the sampling scale (resolution) should always be taken into consideration with caution and the environmental variables and dispersal processes should be distinguished carefully when referring to the effects of temporal and spatial factors (Yeh et al., 2015). On one hand, it is a combination of effect from the aspect of time and space scales because the environmental conditions always change across time and space and may lead to community variation (Fuhrman et al., 2015; Salonen et al., 2019). Thus, different temporal and spatial scales of samples may lead to different conclusions on the processes of community assemblages (Hanson et al., 2012). This study considered community variation in a seasonal scale, which suggests the importance of environmental heterogeneity relating to temporal dynamics in shaping community structure in a relatively small spatial scale (Wu et al., 2017; Salonen et al., 2019). On the other hand, testing the metacommunity theory at multiple scales in a given system needs a corresponding sampling scheme which has enough resolution to include most of the variation in environmental conditions, dispersal barriers, and temporal dynamics with adequate sample sizes (Yeh et al., 2015). For example, the effect of dispersal on boreal lake bacterial communities occurs at very small spatial scale, so that very high-resolution sampling across space is needed (Nino-Garcia et al., 2016b). This is also true at temporal scales where it has been shown that short-term variability in eukaryotes can occur in freshwaters (Mangot et al., 2013), suggesting that a high-resolution sampling over time is needed to estimate the temporal variation of that community. Another potential limitation of this study is the insufficient vertical resolution, for example, the lack of the DCM, especially in summer, when the phenomenon of vertical stratification is obvious (Wang et al., 2012). Potential active and complex microbial interactions may occur in the DCM and lead to specific PCCs and community assembly processes, which need to be explored in future research. In summary, this study makes effort to explain the potential relationship between the PCCs and both of the environmental and spatial factors from multiple spatiotemporal aspects including seasonal variation, habitat heterogeneity, and vertical distribution in waters. However, the statistical results should be interpreted with caution due to small sampling sizes, especially for temporal variation on PCCs for the reason that the sampling points are concentrated in one given month of a single year that may cause poor representativity of seasonal variation. Further studies with enough resolutions on both of the temporal and spatial scales are needed to explore the potential assembly mechanisms of protist community across time and space.

### Other Possible Factors in Shaping Protist Communities

It is notable that the correlations between environmental and spatial factors and PCCs were relatively low and insignificant in most cases, especially for CRT and RT sub-communities. This indicates more complex mechanisms for the determination of PCCs, especially for the rare biosphere. The insignificant and low correlations may be due to unmeasured environmental components (such as other environmental variables that were not measured in this study; Hanson et al., 2012) or other ecological processes (such as neutral process; Bahram et al., 2016), which were overlooked due to the limited sampling effort. Furthermore, other studies indicated that co-occurrence networks within community could also be responsible for the community assembly (Shi et al., 2016; Jiao et al., 2017; Xue et al., 2018). The assembly process of local community can be regarded as the cumulative effects of stochastic processes, environmental selection, and interactions between organisms (HilleRisLambers et al., 2012). Compared with the stochastic and deterministic processes that influence the community assembly in a large scale, biotic interaction within community is considered to be a microscale process that generates predictable patterns of species coexistence in communities (HilleRisLambers et al., 2012).

In the study, we also explore whether the abiotic and biotic variables collected during the three sampled seasons could explain the variations in protists taxa through a constrained ordination analysis (RDA) and investigate the putative interactions between these taxa by inferring correlation networks. On the one hand, all significant correlations among OTUs in the correlation networks were positive, indicating cooperation or co-existence between microorganisms to support the functioning and stability of the ecosystem (Konopka et al., 2015; Shi et al., 2016). The complex and strong associations in the network can be interpreted as multiple biological interactions, such as mutualisms through cross-feeding or exchange of metabolites, antagonism through competition, predation or parasitism, or functional redundancy (Hunt and Ward, 2015). On the other hand, the RDA showed that biological interactions seem to play an important role in the community response to environmental changes, for example, temperature and phosphorus facilitate the growth of phytoplankton production and consequently increase grazing pressure of nanozooplankton (Caron and Hutchins, 2012). Mixotrophic Dinophyceae can feed on bacteria or other smaller protists and can also compete for nutrients with Bacillariophyta, Cryptophyta, or other photosynthetic organisms (Stoecker, 1999). Ciliates have broad resource-use (trophic) categories, acting as predators of bacteria, auto‐ and heterotrophic pico‐ and nanoplankton (Weisse, 2017). The feeding process contributes a lot in the nutrient regeneration (Sherr and Sherr, 2002), which can boost the rate of bacterial growth and further increase the bacterivorous groups. Besides, the MALVs and Apicomplexa have been mostly characterized as parasites that play key roles in controlling the abundance of a wide range of hosts, such as core dinoflagellates (Coats and Park, 2008; Small et al., 2012), radiolarians (Brate et al., 2012), and ciliates (Coats et al., 2012).

The structural and topological properties of the networks reflect the complexity and stability of the community, in response to environmental changes and disturbances (Hunt and Ward, 2015). A relatively looser community structure in the coastal area with low connectivity and more sub-modules supported our findings of the poor consistency of the community-environment relationship in coastal area. Specifically, highly selective environmental conditions in coastal area could weaken the responding of the biological communities, and this lag-back effect would become stronger when the interactions among taxa within the community were looser and weaker as here in coastal area. Correspondingly, the strongly correlated organisms in the shelf area displayed higher environmental sensitivity that tend to respond fast to a relatively gentle environment, leading to a more stable and balanced community. Higher degree of connections of CRT and RT sub-communities compared with CRAT in the biotic associations indicated the importance of rare biosphere which contributes a large part in the whole community. According to the literature, correlation between abundant and rare taxa can enhance the resistance and resilience of the aquatic microbial community when subjected to seasonal environmental variables or subsequent disturbances (Hunt and Ward, 2015; Konopka et al., 2015). Rare biosphere is considered to be irreplaceable in maintaining function and stability of the ecosystem by offering ecological buffering and functional redundancy (Konopka et al., 2015), but of whom the ecological roles are much more overlooked and the biogeographic processes are more complex. Furthermore, the evidence for a rare but active biosphere suggested that the rare taxa do not act only as a seed bank waiting for condition changes, but also exists as an active protist biosphere that contributes to ecosystem functioning (Debroas et al., 2015). In conclusion, the biogeographic processes of protist community assembly depend on the balance of multiple ecological processes such as the external environmental selection, dispersal limitation, drift or other stochastic processes, and endogenous dynamics or interactions within the communities. Therefore, further studies are needed to identify the importance of each of the processes and how they interact to influence the protist community assembly.

## Conclusion

This study provides a comprehensive description on the diversity, community structure, and biogeographic processes of abundant and rare marine protists in the Changjiang plume and its adjacent shelf area, considering scales of time and space. The results supported our hypotheses that the community assembly of PCCs was affected differently by environmental factors, dispersal limitation or other stochastic processes, and biological interactions in different conditions. The highly selective environmental conditions (e.g., temperature, salinity, DIP, Chl *a*, DO, PEUK, HB, and nitrate) due to seasonal dynamics of hydrographical and climatological properties might be the reason for the stronger correlation between environmental factors and PCCs than spatial factors (dispersal limitation) in the studied area. Neutral model suggested a predominant role of neutral process (dispersal, drift, etc.) in shaping the PCCs in the coastal area. Co-occurrence network analysis further exhibited a complex and unstable community with low connectivity and more sub-modules in the coastal area. Correspondingly, the shelf area exhibited a better consistency of the community-environment relationship, stronger correlation coefficients between environmental factors and PCCs, higher contribution of spatial factors (dispersal limitation) on PCCs, and a more stable and diverse protist community. In addition, the abundant sub-communities exhibited higher dispersal ability which tend to respond to environmental selection during dispersal, whereas the rare sub-communities appeared to be present only in few samples due to dispersal limitation. Altogether, our results emphasize the importance of considering environmental factors, spatial factors, and biotic interactions when determining the protist community assembly, and highlight the potential roles of rare taxa or conditionally rare taxa in community structure and functioning. However, a much greater degree of temporal resolution is needed to better understand how seasonal fluctuations regulate the dynamics and structuring of PCCs.

## Data Availability Statement

The datasets presented in this study can be found in online repositories. The names of the repository/repositories and accession number(s) can be found below: https://www.ncbi.nlm.nih.gov/, PRJNA551318.

## Author Contributions

LH and LW designed the experiment. LW collected the samples and conducted the experiments. XG analyzed the data and wrote the article. All authors contributed to the article and approved the submitted version.

### Conflict of Interest

The authors declare that the research was conducted in the absence of any commercial or financial relationships that could be construed as a potential conflict of interest.

## Footnotes

^1^http://www.mothur.org.\/wiki/454_SOP

^2^http://www.arb-silva.de/

^3^http://www.gephi.org/
