# Supplementary material for: Spatiotemporal Patterns in Diversity and Assembly Process of Marine Protist Communities of the Changjiang (Yangtze River) Plume and Its Adjacent Waters
Source: Front Microbiol. 2020 Oct 6;11:579290. doi: 10.3389/fmicb.2020.579290 (PMC7573215; doi:10.3389/fmicb.2020.579290)
Supplement: Supplementary file 1 [file Data_Sheet_1.docx]

Supplementary Material

**~~Spatiotemporal patterns of diversity and biogeography of marine protist communities off the Changjiang (Yangtze River) plume and its adjacent waters~~**

**Spatiotemporal patterns in diversity and assembly process of marine protist communities of the Changjiang (Yangtze River) plume and its adjacent waters**

**Xin Guo^1^, Linnan Wu^1^, Lingfeng Huang^1 *^**

^1^ Key Laboratory of the Ministry of Education for Coastal and Wetland Ecosystems, College of the Environment and Ecology, Xiamen University, Xiamen, 361102, China

*** Correspondence:**Lingfeng Huang
huanglf@xmu.edu.cn

**The supplementary information includes:**

- **18 pages**
- **9 supplementary figures (pages 2-10)**
- **6 supplementary tables (pages 11-18)**

**Supplemental Figures**

**Supplementary Fig. S1** Rarefaction curves of similarity-based operational taxonomic unit (OTUs) at 97 % sequence identity threshold. **A.** The individual 60 samples. **B.** The combined sets of each of three months samples (20 samples in each of the three months). **C.** The combined sets of each of six months-sites groups samples (10 samples in May-coast, May-shelf, Aug-coast and Aug-shelf groups, 12 samples in Oct-coast and 8 samples in Oct-shelf). **D.** The combined set of 60 samples.


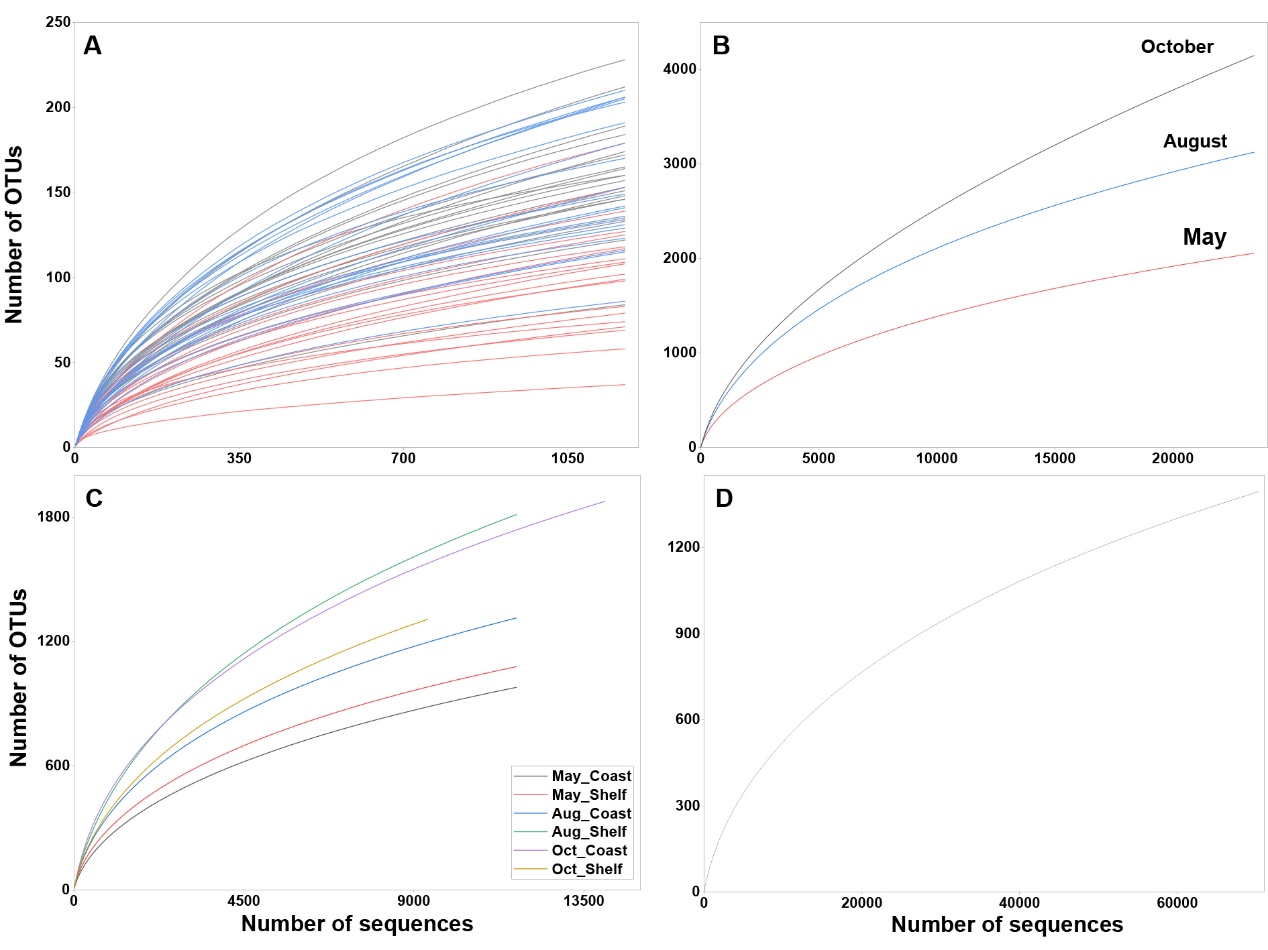


**Supplementary Fig. S2** Boxplots displayed the alpha-diversity of protists communities (Sobs was the observed OTU number, ACE, Chao1, Shannon, Simpson and Pielou evenness) in surface and bottom layers. Non-parametric test (Mann-Whitney U test) was used to compare the differences and all the *P* values were large than 0.1. The hollow diamonds represented average values of individual index in each group.


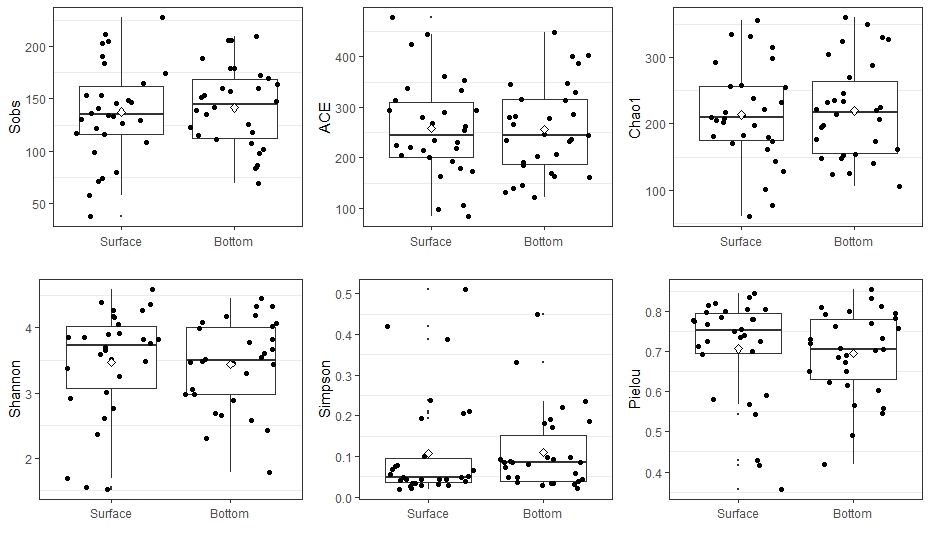


**Supplementary Fig. S3** Relative contributions of richness (OTUs) and abundance (reads) of the taxonomic groups in the CRAT, CRT, and RT sub-communities


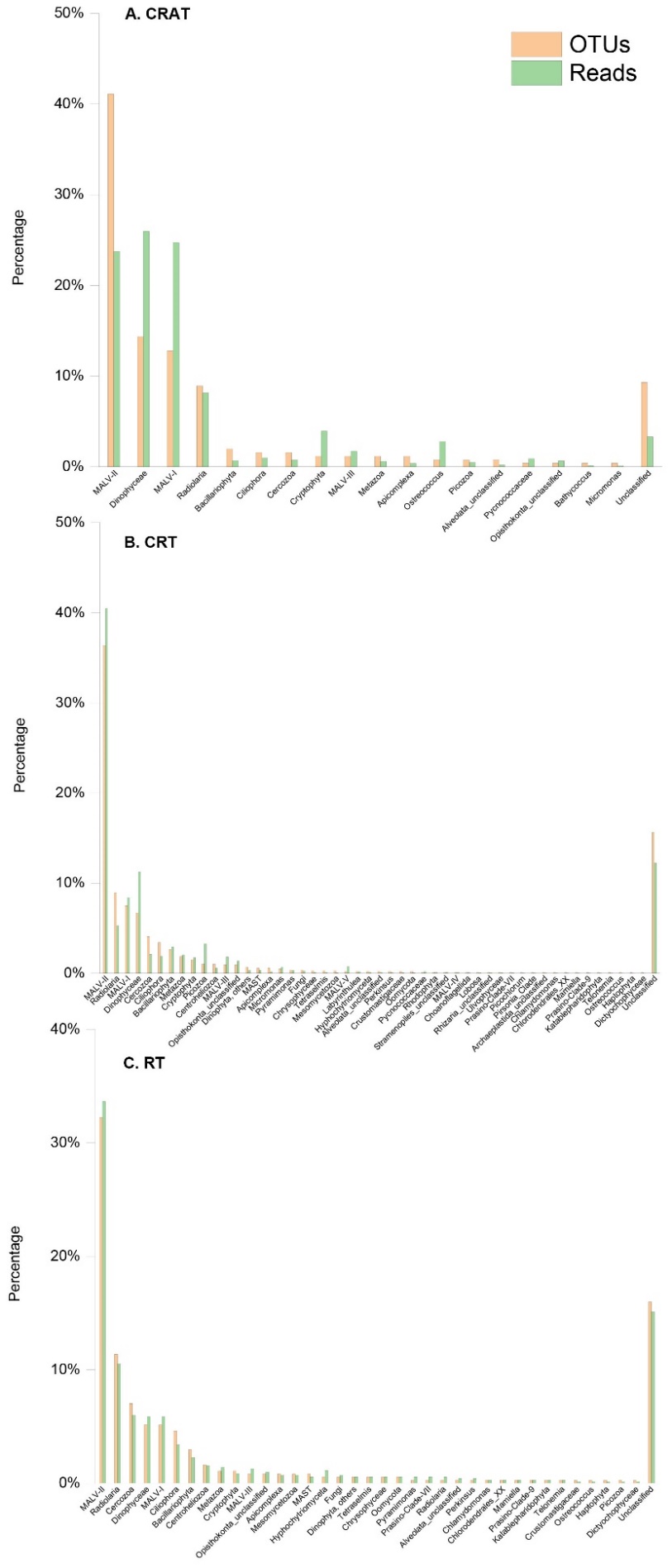


**Supplementary Fig. S4** Distribution of abundance of conditionally rare and abundant taxa (CRAT) in each of the 60 samples. (**A**) Heatmap indicated whether CRAT taxa (vertical lines) were abundant (> 1 %; black) or rare (< 0.01 %; white) or had intermediate (gray) abundance in specific samples. Dendrogram of 60 samples with furthest neighbor clustering method of CRAT sub-community was based on squared Euclidean distance. (**B**) Histograms indicated the number of samples in which CRAT were abundant or rare or intermediate in each data set. Some OTUs of CRAT tended to be abundant in a single sample or two samples, while some OTUs were rare in at least one fifth of total samples. (**C**) Frequency distribution of all taxa from 60 samples.


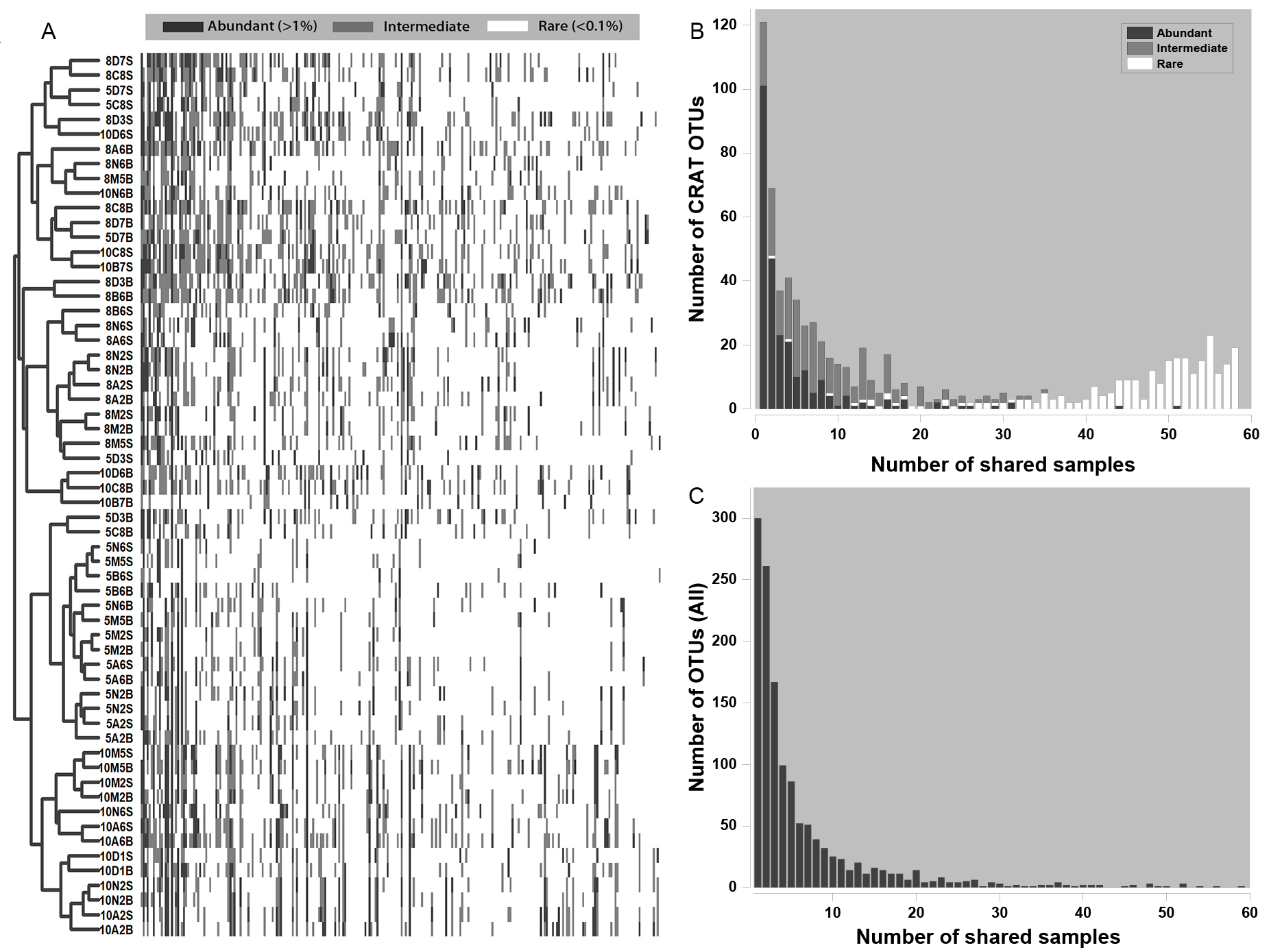


**Supplementary Fig. S5** Venn diagrams showing the unique and shared OTUs of 1 332 all OTUs and three microbial subcommunities (258 CRAT OTUs, 1 053 CRT OTUs and 369 RT OTUs) in three months, two layers and two habitats. The operational taxonomic units (OTUs) were defined at 97 % sequence similarity threshold.


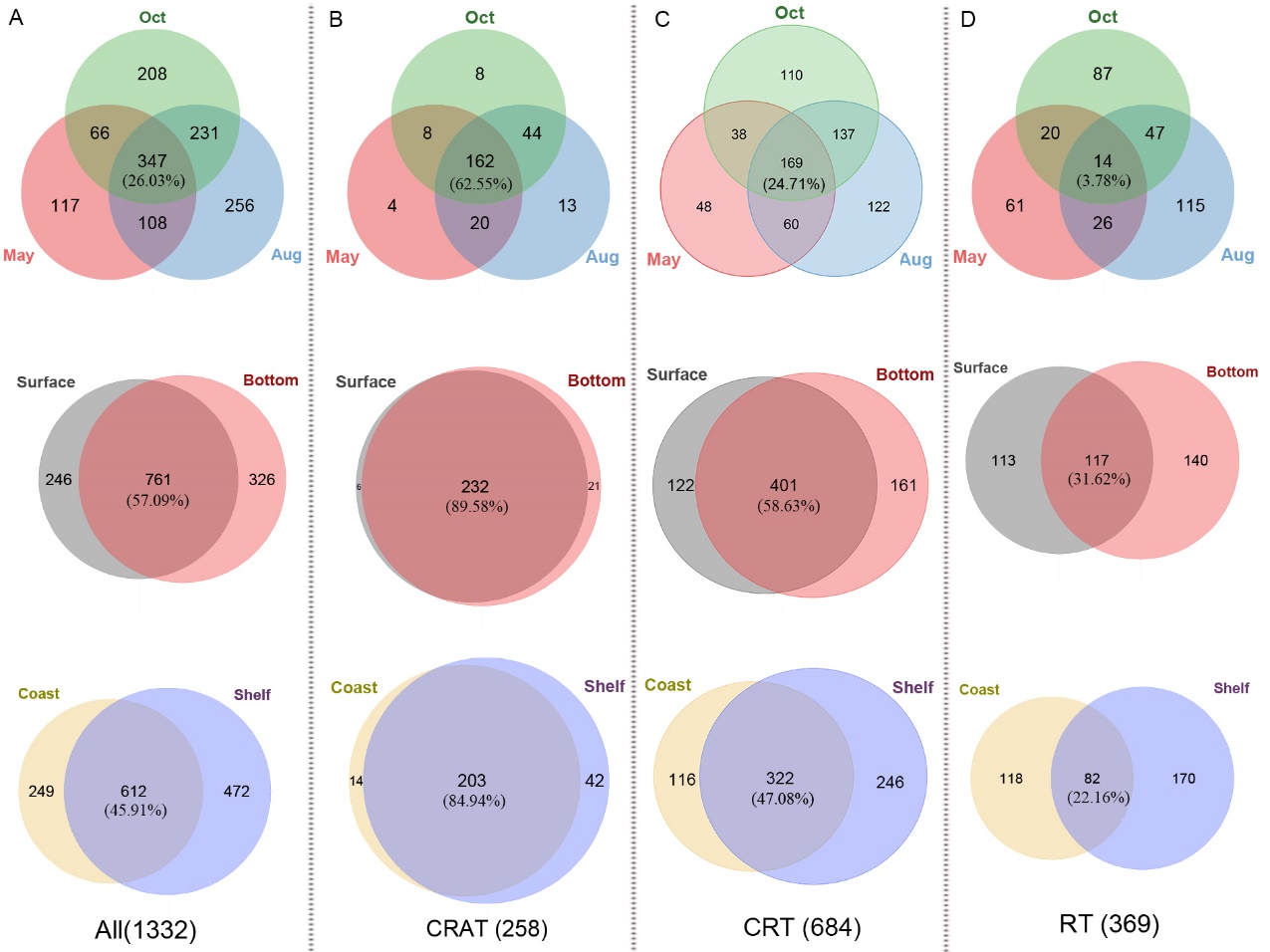


**Supplementary Fig. S6** Distance-decay patterns of the protist communities based on the Mantel tests for the correlations between the Bray-Curtis similarity (1 - Bray-Curtis distance) of protist community composition, geographic distance, and the Euclidean distance of environmental factors in all samples using Spearman's rank coefficients. Lines represent linear fits.


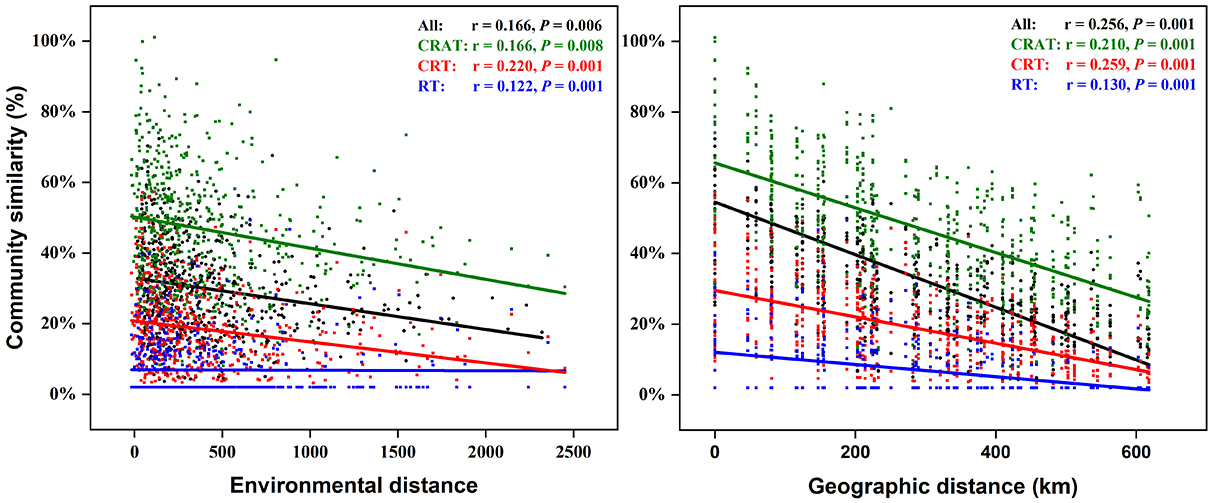


**Supplementary Fig. S7** Spearman’s rank correlation between the Euclidean distance of environmental variables and geographic distance.


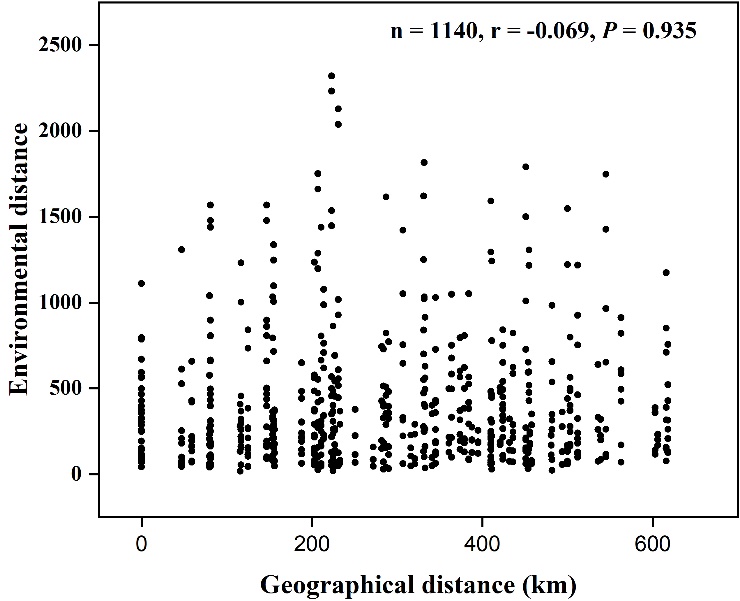


**Supplementary Fig. S8** Mantel test for the correlations among environmental distance (E), spatial distance (S), and protist communities’ distance in three seasons (**A**) and two layers (**B**) using Spearman's rank coefficients.


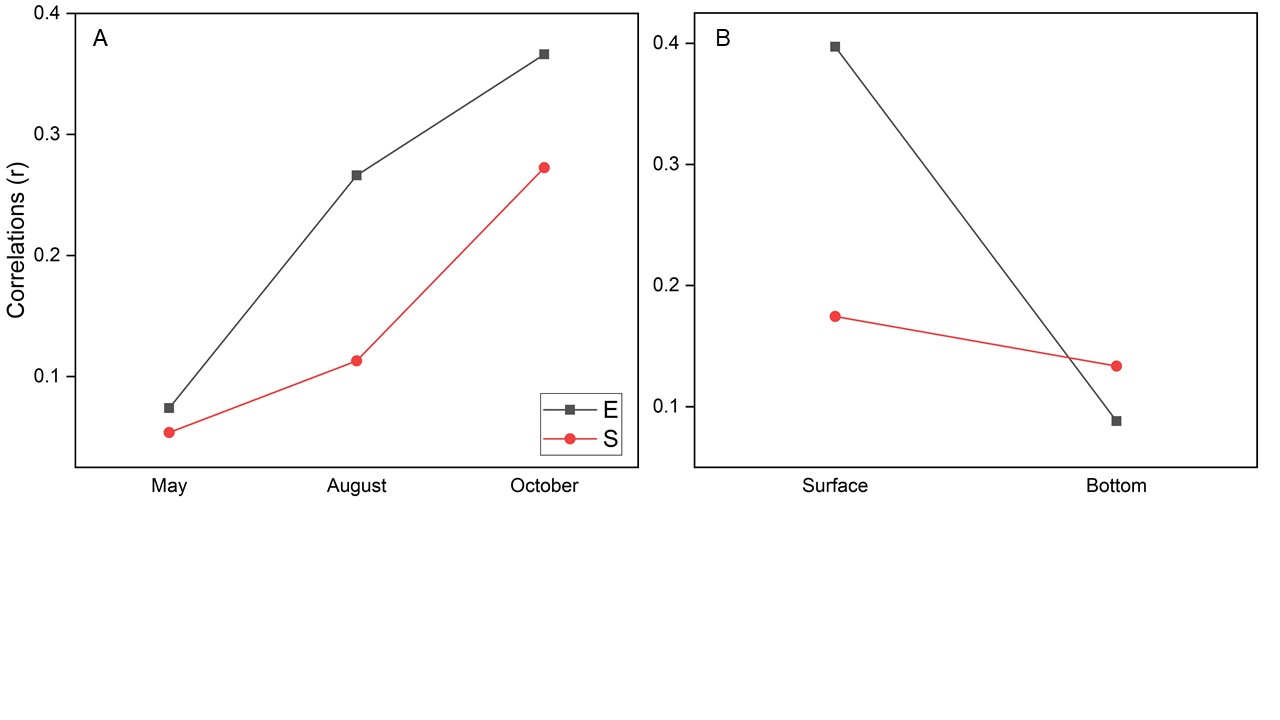


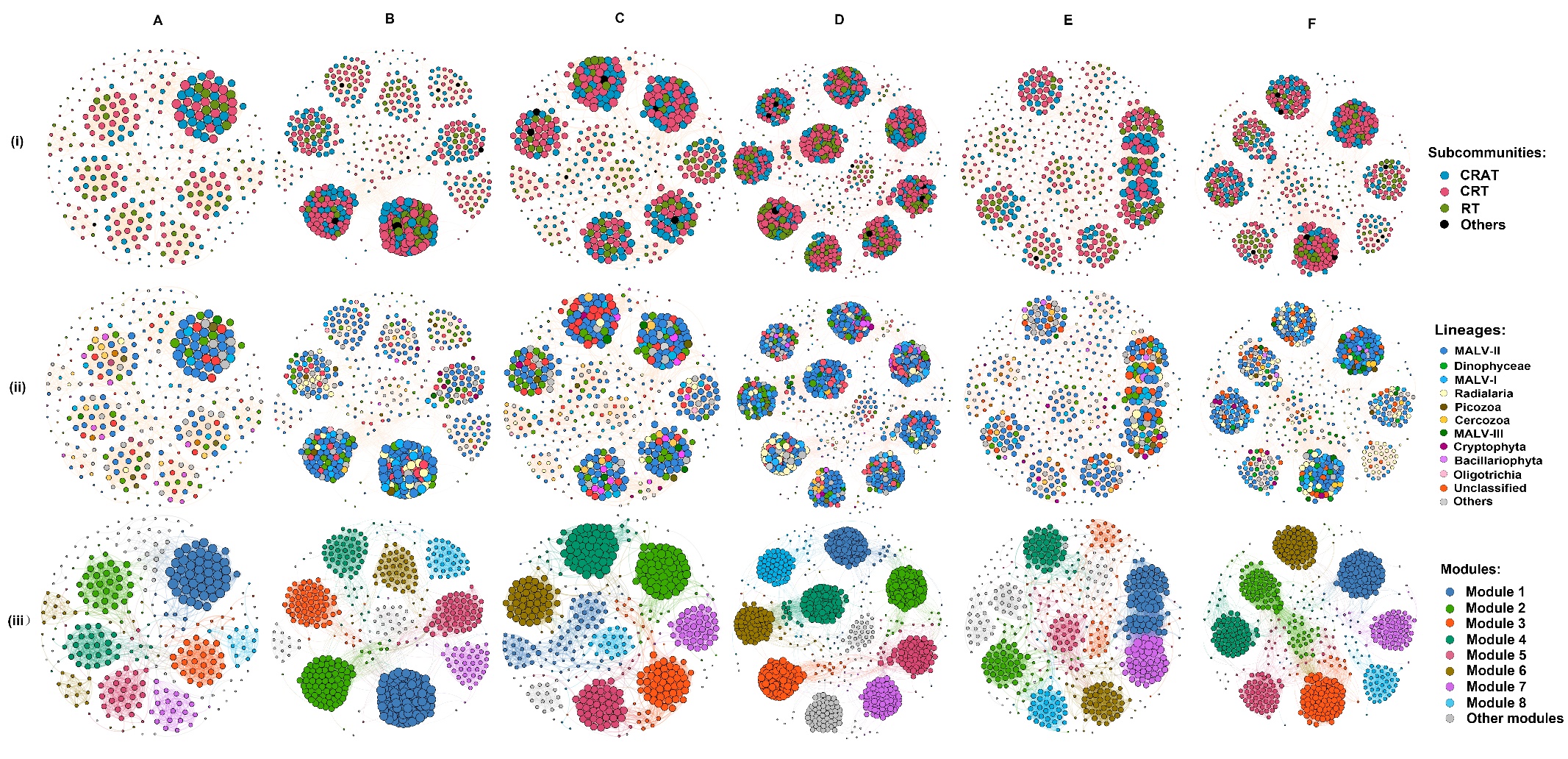


**Supplementary Fig. S9** Co-occurrence networks among OTUs of protist community in six sampling groups (**A.** May-Coast, **B.** May-Shelf, **C.** Aug-Coast, **D.** Aug-Shelf, **E.** Oct-Coast, **F.** Oct-Shelf). The nodes were colored according to different categories of sub-communities (**i**), taxonomic lineages (**ii**) and modules (**iii**). Connections between two individual nodes stand for strong (Spearman’s r > 0.6 or r < -0.6) and significant (*P* < 0.05) correlation. The size of each node is proportional to the number of connections (i.e., degree).

**Supplemental Tables**

**Supplementary Table S1** Sample list and the abundance, richness and diversity of protist communities in each individual sample. Depth (m), the sampling layer; Bot_depth (m), the depth of the station; NP, nano-sized protists (cells/mL); HNP, heterotrophic nano-sized protists (cells/mL); PNP, pigmented nano-sized protists (cells/mL); size_2_5, size fraction of 2-5 μm of NP (cells/mL); size_5_10, size fraction of 5-10 μm of NP (cells/mL); size_10_20, size fraction of 10-20 μm of NP (cells/mL). Samples are labelled with the sampling months (5 for May, 8 for August, 10 for October) and the sampling layers (B for bottom, S for surface) and are grouped into six groups based on sampling months and habitats.

| **Sample** | **Longitude** | **Latitude** | **Depth** | **Bot_Depth** | **Groups** | **NP** | **HNP** | **PNP** | **size_2_5** | **size_5_10** | **size_10_20** | **OTUs** | **ACE** | **Chao1** | **Shannon** | **Simpson** | **Pielou** | **Coverage** |
| --- | --- | --- | --- | --- | --- | --- | --- | --- | --- | --- | --- | --- | --- | --- | --- | --- | --- | --- |
| 5A2B | 122.64 | 31.64 | 30 | 30 | May-Coast | 1056 | 741 | 315 | 815 | 204 | 37 | 118 | 163 | 161 | 2.97 | 0.17 | 0.62 | 0.96 |
| 5A2S | 122.64 | 31.64 | 2 | 30 | May-Coast | 1537 | 759 | 778 | 759 | 685 | 93 | 109 | 204 | 183 | 3.25 | 0.08 | 0.69 | 0.96 |
| 5A6B | 124.75 | 32.00 | 42 | 42 | May-Coast | 1574 | 796 | 778 | 1185 | 370 | 19 | 83 | 122 | 124 | 2.66 | 0.19 | 0.60 | 0.97 |
| 5A6S | 124.75 | 32.00 | 2 | 42 | May-Coast | 1074 | 722 | 352 | 759 | 296 | 19 | 117 | 279 | 217 | 2.77 | 0.19 | 0.58 | 0.95 |
| 5B6B | 125.79 | 31.03 | 62 | 62 | May-Shelf | 1667 | 1167 | 500 | 1334 | 222 | 111 | 98 | 145 | 153 | 2.98 | 0.09 | 0.65 | 0.97 |
| 5B6S | 125.79 | 31.03 | 2 | 62 | May-Shelf | 2556 | 2334 | 222 | 2334 | 185 | 37 | 37 | 98 | 60 | 1.55 | 0.39 | 0.43 | 0.99 |
| 5C8B | 125.62 | 28.99 | 104 | 104 | May-Shelf | 815 | 463 | 352 | 111 | 222 | 482 | 111 | 162 | 154 | 3.31 | 0.08 | 0.70 | 0.96 |
| 5C8S | 125.62 | 28.99 | 2 | 104 | May-Shelf | 1223 | 1019 | 204 | 982 | 185 | 56 | 127 | 180 | 174 | 3.76 | 0.04 | 0.78 | 0.96 |
| 5D3B | 122.63 | 29.06 | 56 | 56 | May-Shelf | 963 | 704 | 259 | 852 | 56 | 56 | 139 | 207 | 194 | 3.61 | 0.06 | 0.73 | 0.95 |
| 5D3S | 122.63 | 29.06 | 2 | 56 | May-Shelf | 5557 | 2945 | 2612 | 5446 | 74 | 37 | 74 | 105 | 101 | 3.01 | 0.10 | 0.70 | 0.98 |
| 5D7B | 125.07 | 27.67 | 104 | 104 | May-Shelf | 352 | 333 | 19 | 204 | 148 | 0 | 179 | 282 | 269 | 3.66 | 0.10 | 0.71 | 0.93 |
| 5D7S | 125.07 | 27.67 | 2 | 104 | May-Shelf | 370 | 352 | 19 | 259 | 37 | 74 | 153 | 361 | 299 | 3.86 | 0.04 | 0.77 | 0.94 |
| 5M2B | 122.52 | 32.76 | 21 | 21 | May-Coast | 500 | 167 | 333 | 482 | 19 | 0 | 69 | 140 | 105 | 1.77 | 0.45 | 0.42 | 0.97 |
| 5M2S | 122.52 | 32.76 | 2 | 21 | May-Coast | 1167 | 482 | 685 | 982 | 167 | 19 | 79 | 201 | 143 | 2.37 | 0.24 | 0.54 | 0.97 |
| 5M5B | 123.10 | 32.97 | 31 | 31 | May-Coast | 778 | 482 | 296 | 741 | 37 | 0 | 108 | 267 | 177 | 2.30 | 0.33 | 0.49 | 0.95 |
| 5M5S | 123.10 | 32.97 | 2 | 31 | May-Coast | 1167 | 834 | 333 | 963 | 204 | 0 | 71 | 164 | 128 | 1.52 | 0.51 | 0.36 | 0.97 |
| 5N2B | 122.51 | 32.05 | 27 | 27 | May-Coast | 945 | 167 | 778 | 796 | 148 | 0 | 125 | 246 | 174 | 3.47 | 0.08 | 0.72 | 0.96 |
| 5N2S | 122.51 | 32.05 | 2 | 27 | May-Coast | 1352 | 204 | 1148 | 1000 | 352 | 0 | 99 | 174 | 162 | 2.61 | 0.21 | 0.57 | 0.96 |
| 5N6B | 124.74 | 32.73 | 60 | 60 | May-Shelf | 630 | 537 | 93 | 482 | 130 | 19 | 102 | 191 | 147 | 2.58 | 0.19 | 0.56 | 0.96 |
| 5N6S | 124.74 | 32.73 | 2 | 60 | May-Shelf | 1482 | 926 | 556 | 1148 | 333 | 0 | 58 | 83 | 77 | 1.69 | 0.42 | 0.42 | 0.98 |
| 8A2B | 122.64 | 31.64 | 30 | 34 | Aug-Coast | - | - | - | - | - | - | 153 | 402 | 351 | 3.44 | 0.09 | 0.68 | 0.93 |
| 8A2S | 122.64 | 31.64 | 2 | 34 | Aug-Coast | - | - | - | - | - | - | 149 | 254 | 208 | 3.90 | 0.04 | 0.78 | 0.95 |
| 8A6B | 124.75 | 32.00 | 43 | 45 | Aug-Coast | - | - | - | - | - | - | 170 | 233 | 235 | 4.08 | 0.04 | 0.80 | 0.95 |
| 8A6S | 124.75 | 32.00 | 2 | 45 | Aug-Coast | - | - | - | - | - | - | 131 | 193 | 198 | 3.66 | 0.05 | 0.75 | 0.96 |
| 8B6B | 125.79 | 31.03 | 62 | 62 | Aug-Shelf | 1074 | 926 | 148 | 796 | 167 | 111 | 206 | 449 | 361 | 4.06 | 0.05 | 0.76 | 0.91 |
| 8B6S | 125.79 | 31.03 | 2 | 62 | Aug-Shelf | 1704 | 1056 | 648 | 1426 | 204 | 74 | 141 | 295 | 213 | 2.92 | 0.21 | 0.59 | 0.94 |
| 8C8B | 125.62 | 28.99 | 105 | 105 | Aug-Shelf | 537 | 519 | 19 | 315 | 37 | 185 | 210 | 315 | 330 | 4.45 | 0.02 | 0.83 | 0.92 |
| 8C8S | 125.62 | 28.99 | 2 | 105 | Aug-Shelf | 1148 | 1074 | 74 | 778 | 222 | 148 | 203 | 290 | 257 | 4.27 | 0.03 | 0.80 | 0.94 |
| 8D3B | 122.63 | 29.06 | 54 | 54 | Aug-Shelf | 926 | 704 | 222 | 741 | 111 | 74 | 206 | 404 | 324 | 4.33 | 0.04 | 0.81 | 0.92 |
| 8D3S | 122.63 | 29.06 | 2 | 54 | Aug-Shelf | 2112 | 1426 | 685 | 1704 | 333 | 74 | 205 | 478 | 355 | 4.36 | 0.03 | 0.82 | 0.92 |
| 8D7B | 125.07 | 27.67 | 98 | 98 | Aug-Shelf | 389 | 296 | 93 | 296 | 93 | 0 | 179 | 386 | 327 | 3.49 | 0.09 | 0.67 | 0.92 |
| 8D7S | 125.07 | 27.67 | 2 | 98 | Aug-Shelf | 963 | 889 | 74 | 704 | 222 | 37 | 191 | 295 | 293 | 4.38 | 0.02 | 0.83 | 0.93 |
| 8M2B | 122.52 | 32.76 | 23 | 23 | Aug-Coast | 1334 | 1074 | 259 | 852 | 296 | 185 | 86 | 132 | 125 | 2.43 | 0.22 | 0.55 | 0.97 |
| 8M2S | 122.52 | 32.76 | 2 | 23 | Aug-Coast | 2167 | 1093 | 1074 | 1148 | 463 | 556 | 116 | 267 | 222 | 3.38 | 0.07 | 0.71 | 0.95 |
| 8M5B | 123.10 | 32.97 | 31 | 31 | Aug-Coast | 1278 | 741 | 537 | 667 | 389 | 222 | 115 | 244 | 207 | 2.68 | 0.23 | 0.56 | 0.95 |
| 8M5S | 123.10 | 32.97 | 2 | 31 | Aug-Coast | 3093 | 1056 | 2038 | 2075 | 556 | 463 | 134 | 222 | 179 | 3.69 | 0.05 | 0.75 | 0.96 |
| 8N2B | 122.51 | 32.05 | 26 | 26 | Aug-Coast | 1111 | 926 | 185 | 963 | 111 | 37 | 123 | 169 | 147 | 3.51 | 0.07 | 0.73 | 0.97 |
| 8N2S | 122.51 | 32.05 | 2 | 26 | Aug-Coast | 1148 | 945 | 204 | 982 | 148 | 19 | 136 | 235 | 205 | 3.82 | 0.04 | 0.78 | 0.95 |
| 8N6B | 124.74 | 32.73 | 60 | 60 | Aug-Shelf | 1093 | 1056 | 37 | 1000 | 93 | 0 | 142 | 286 | 220 | 3.05 | 0.18 | 0.62 | 0.95 |
| 8N6S | 124.74 | 32.73 | 2 | 60 | Aug-Shelf | 1408 | 1204 | 204 | 1037 | 296 | 74 | 129 | 215 | 180 | 3.52 | 0.07 | 0.72 | 0.96 |
| 10A2B | 122.64 | 31.64 | 30 | 30 | Oct-Coast | 352 | 278 | 74 | 241 | 111 | 0 | 172 | 330 | 245 | 4.17 | 0.03 | 0.81 | 0.94 |
| 10A2S | 122.64 | 31.64 | 2 | 30 | Oct-Coast | 1111 | 1000 | 111 | 1000 | 93 | 19 | 165 | 314 | 232 | 4.15 | 0.03 | 0.81 | 0.94 |
| 10A6B | 124.75 | 32.00 | 42 | 44 | Oct-Coast | - | - | - | - | - | - | 189 | 315 | 305 | 4.20 | 0.03 | 0.80 | 0.92 |
| 10A6S | 124.75 | 32.00 | 2 | 44 | Oct-Coast | - | - | - | - | - | - | 184 | 353 | 255 | 4.17 | 0.03 | 0.80 | 0.93 |
| 10B7B | 126.89 | 30.96 | 97 | 97 | Oct-Shelf | - | - | - | - | - | - | 84 | 186 | 141 | 2.88 | 0.10 | 0.65 | 0.97 |
| 10B7S | 126.89 | 30.96 | 2 | 97 | Oct-Shelf | - | - | - | - | - | - | 212 | 444 | 335 | 4.26 | 0.04 | 0.80 | 0.92 |
| 10C8B | 125.62 | 28.99 | 105 | 105 | Oct-Shelf | 222 | 185 | 37 | 167 | 37 | 19 | 164 | 346 | 288 | 3.99 | 0.03 | 0.78 | 0.94 |
| 10C8S | 125.62 | 28.99 | 2 | 105 | Oct-Shelf | 370 | 278 | 93 | 111 | 204 | 56 | 228 | 333 | 332 | 4.58 | 0.02 | 0.84 | 0.92 |
| 10D1B | 122.32 | 29.21 | 17 | 17 | Oct-Coast | 296 | 241 | 56 | 241 | 56 | 0 | 157 | 280 | 232 | 3.82 | 0.05 | 0.76 | 0.95 |
| 10D1S | 122.32 | 29.21 | 2 | 17 | Oct-Coast | 3130 | 2241 | 889 | 2853 | 241 | 37 | 133 | 261 | 202 | 3.59 | 0.05 | 0.73 | 0.95 |
| 10D6B | 125.50 | 27.95 | 100 | 100 | Oct-Shelf | 259 | 259 | 0 | 204 | 56 | 0 | 148 | 345 | 234 | 3.45 | 0.08 | 0.69 | 0.94 |
| 10D6S | 125.50 | 27.95 | 2 | 100 | Oct-Shelf | 2186 | 1963 | 222 | 2056 | 111 | 19 | 174 | 425 | 316 | 3.82 | 0.05 | 0.74 | 0.92 |
| 10M2B | 122.44 | 32.72 | 23 | 26 | Oct-Coast | - | - | - | - | - | - | 160 | 234 | 221 | 4.02 | 0.03 | 0.79 | 0.95 |
| 10M2S | 122.44 | 32.72 | 2 | 26 | Oct-Coast | - | - | - | - | - | - | 122 | 225 | 171 | 3.48 | 0.07 | 0.72 | 0.96 |
| 10M5B | 123.10 | 32.97 | 31 | 31 | Oct-Coast | 778 | 630 | 148 | 519 | 167 | 93 | 160 | 202 | 213 | 4.33 | 0.02 | 0.85 | 0.96 |
| 10M5S | 123.10 | 32.97 | 2 | 31 | Oct-Coast | 1630 | 1426 | 204 | 1278 | 278 | 74 | 153 | 337 | 258 | 4.05 | 0.03 | 0.81 | 0.94 |
| 10N2B | 122.51 | 32.05 | 24 | 24 | Oct-Coast | 630 | 611 | 19 | 445 | 130 | 56 | 135 | 237 | 198 | 3.78 | 0.04 | 0.77 | 0.95 |
| 10N2S | 122.51 | 32.05 | 2 | 24 | Oct-Coast | 722 | 611 | 111 | 482 | 241 | 0 | 146 | 231 | 239 | 3.91 | 0.03 | 0.79 | 0.95 |
| 10N6B | 124.74 | 32.73 | 60 | 60 | Oct-Shelf | 648 | 593 | 56 | 445 | 111 | 93 | 151 | 279 | 225 | 3.54 | 0.08 | 0.71 | 0.94 |
| 10N6S | 124.74 | 32.73 | 2 | 60 | Oct-Shelf | 2445 | 2223 | 222 | 2186 | 222 | 37 | 146 | 218 | 210 | 3.85 | 0.04 | 0.77 | 0.95 |
| **All samples** |  |  |  |  |  | **1261** | **873** | **388** | **987** | **198** | **76** | **1332** | **1429** | **1412** | **5.21** | **0.02** | **0.72** |  |

**Supplementary Table S2** General descriptions of All, CRAT, CRT, and RT microbial taxa categories to protists community in the combined set of 60 samples.

| Categories of the communities | OTUs number | Sequence number | Chao1 | ACE |
| --- | --- | --- | --- | --- |
| All OTUs (All) | 1332 | 70320 | 1412 ± 16 | 1429 ± 18 |
| Abundant taxa (AT) | 0 | 0 | - | - |
| Conditionally abundant taxa (CAT) | 0 | 0 | - | - |
| Moderate taxa (MT) | 0 | 0 | - | - |
| Conditionally rare and abundant taxa (CRAT) | 258 (19.37 %) | 61516 (87.48 %) | 258 | NA |
| Conditionally rare taxa (CRT) | 684 (51.35 %) | 7508 (10.68 %) | 684 | 684 ± 12 |
| Rare taxa (RT) | 369 (27.70 %) | 701 (1.00 %) | 490 ± 24 | 518 ± 13 |

**Supplementary Table S3** Two-way analysis of variance showing the effects of seasonality and/or habitats (Coast and Shelf area) on the α-diversity estimators of operational taxonomic units (97% similarity cut-off) of the CRAT, CRT, and RT sub-communities.

| **Diversity** | **Seasons** | | | | | |  | **Habitats** | | | | | |  | **Seasons × Habitats** | | | | | |
| --- | --- | --- | --- | --- | --- | --- | --- | --- | --- | --- | --- | --- | --- | --- | --- | --- | --- | --- | --- | --- |
|  | **CRAT** | | **CRT** | | **RT** | |  | **CRAT** | | **CRT** | | **RT** | |  | **CRAT** | | **CRT** | | **RT** | |
|  | **F** | ***P*** | **F** | ***P*** | **F** | ***P*** |  | **F** | ***P*** | **F** | ***P*** | **F** | ***P*** |  | **F** | ***P*** | **F** | ***P*** | **F** | ***P*** |
| **Sobs** | **17.77** | **0.00** | **21.44** | **0.00** | **11.74** | **0.00** |  | 1.79 | 0.19 | **12.97** | **0.00** | **11.61** | **0.00** |  | 1.47 | 0.24 | **5.47** | **0.01** | **7.89** | **0.00** |
| **ACE** | **8.47** | **0.00** | **11.95** | **0.00** |  |  |  | 1.25 | 0.27 | 3.25 | 0.08 |  |  |  | 2.41 | 0.10 | 3.20 | 0.05 |  |  |
| **Chao1** | **7.35** | **0.00** | **11.55** | **0.00** | **9.10** | **0.00** |  | 0.81 | 0.37 | 3.33 | 0.07 | **16.08** | **0.00** |  | 1.61 | 0.21 | 2.41 | 0.10 | **8.11** | **0.00** |
| **Shannon** | **16.91** | **0.00** | **16.43** | **0.00** | **14.34** | **0.00** |  | 0.43 | 0.52 | 2.95 | 0.09 | 3.91 | 0.05 |  | 2.19 | 0.12 | **4.31** | **0.02** | **7.31** | **0.00** |
| **Simpson** | **13.17** | **0.00** | **5.35** | **0.01** | **11.65** | **0.00** |  | 0.91 | 0.34 | 0.04 | 0.85 | 0.00 | 1.00 |  | 2.20 | 0.12 | 1.84 | 0.17 | **5.78** | **0.01** |
| **Pielou‘s** | **14.86** | **0.00** | 0.35 | 0.71 |  |  |  | 0.26 | 0.62 | 2.54 | 0.12 |  |  |  | 2.44 | 0.10 | 2.63 | 0.08 |  |  |
| Significant *P*-values (< 0.05) are highlighted in bold. F, F-statistic; *P*, *P*-values. | | | | | | | | | | | | | | | | | | | | |

**Supplementary Table S4** Analysis of similarities (ANOSIM) results of different comparisons based on months (May, August and October), layers (B for bottom and S for surface) and sampling sites (Coast and Shelf) among CRT, RT, and CRAT microbial taxa categories to protists community based on Bray-curtis distance.

| **Distance between samples** | | **Bray-Curtis** | | | | | | | |
| --- | --- | --- | --- | --- | --- | --- | --- | --- | --- |
| **Microbial taxa category** | | **RT** | |  | **CRT** | |  | **CRAT** | |
| **Grouping by** | **Comparisons** | **R-value** | ***P*-value** |  | **R-value** | ***P*-value** |  | **R-value** | ***P*-value** |
| **Month** | Aug vs. May | 0.169 | < 0.001* |  | 0.432 | < 0.001* |  | 0.452 | < 0.001* |
|  | Aug vs. Oct | 0.222 | < 0.001* |  | 0.286 | < 0.001* |  | 0.343 | < 0.001* |
|  | May vs. Oct | 0.192 | < 0.001* |  | 0.410 | < 0.001* |  | 0.514 | < 0.001* |
|  | Aug_B vs. May_B | **0.027** | **0.317** |  | 0.401 | 0.001* |  | 0.418 | < 0.001* |
|  | **Aug_B vs. Oct_B** | **0.083** | **0.108** |  | **0.117** | **0.071** |  | 0.218 | 0.004* |
|  | Aug_S vs. May_S | 0.251 | < 0.001* |  | 0.430 | < 0.001* |  | 0.513 | < 0.001* |
|  | Aug_S vs. Oct_S | 0.294 | < 0.001* |  | 0.515 | < 0.001* |  | 0.534 | < 0.001* |
|  | May_B vs. Oct_B | 0.116 | 0.033 |  | 0.403 | < 0.001* |  | 0.362 | 0.002* |
|  | May_S vs. Oct_S | 0.211 | 0.001* |  | 0.468 | < 0.001* |  | 0.728 | < 0.001* |
|  | Aug_Coast vs. May_Coast | 0.412 | < 0.001* |  | 0.889 | < 0.001* |  | 0.854 | < 0.001* |
|  | Aug_Coast vs. Oct_Coast | 0.526 | < 0.001* |  | 0.761 | < 0.001* |  | 0.806 | < 0.001* |
|  | May_Coast vs. Oct_Coast | 0.446 | < 0.001* |  | 0.950 | < 0.001* |  | 0.978 | < 0.001* |
|  | Aug_Shelf vs. May_Shelf | **0.050** | **0.191** |  | 0.235 | 0.004* |  | 0.228 | 0.002* |
|  | **Aug_Shelf vs. Oct_Shelf** | **0.038** | **0.312** |  | **0.085** | **0.140** |  | **0.074** | **0.155** |
|  | May_Shelf vs. Oct_Shelf | **0.068** | **0.151** |  | 0.163 | 0.038 |  | 0.260 | 0.002* |
| **Layer** | B vs. S | 0.035 | 0.033* |  | 0.149 | < 0.001* |  | 0.120 | < 0.001* |
|  | Aug_B vs. Aug_S | **0.022** | **0.353** |  | 0.274 | 0.006* |  | 0.234 | 0.003* |
|  | **May_B vs. May_S** | **0.010** | **0.410** |  | 0.114 | 0.047 |  | **0.052** | **0.139** |
|  | **Oct_B vs. Oct_S** | **-0.011** | **0.499** |  | **0.078** | **0.095** |  | **0.049** | **0.141** |
|  | **Coast_B vs. Coast_S** | **-0.025** | **0.742** |  | **-0.024** | **0.672** |  | **0.009** | **0.328** |
|  | Shelf_B vs. Shelf_S | 0.160 | 0.001* |  | 0.446 | < 0.001* |  | 0.288 | < 0.001* |
| **Habitats** | Coast vs. Shelf | 0.139 | < 0.001* |  | 0.254 | < 0.001* |  | 0.219 | < 0.001* |
|  | Aug_Coast vs. Aug_Shelf | 0.237 | 0.001* |  | 0.412 | 0.001* |  | 0.335 | < 0.001* |
|  | May_Coast vs. May_Shelf | 0.162 | 0.007* |  | 0.360 | < 0.001* |  | 0.318 | 0.002* |
|  | Oct_Coast vs. Oct_Shelf | 0.422 | < 0.001* |  | 0.691 | < 0.001* |  | 0.575 | < 0.001* |
|  | Coast_B vs. Shelf_B | 0.197 | < 0.001* |  | 0.361 | < 0.001* |  | 0.307 | < 0.001* |
|  | Coast_S vs. Shelf_S | 0.103 | 0.013 |  | 0.239 | < 0.001* |  | 0.174 | 0.003* |
| **Bold** fonds represented **insignificant** difference with *P* values higher than 0.05. | | | | | | | | | |

**Supplementary Table S5** Correlation analyses among the environmental factors (including abiotic and biotic factors) and the abundance and diversity of protists communities based on Spearman coefficient. Refer to Fig. 1 and Supplemental Table S1 for variable abbreviations.

| **Correlation (r)** | **Depth** | **Bot_**  **depth** | **Temp** | **Sal** | **DO** | **NH_4_-N** | **NO_3_-N** | **NO_2_-N** | **DIP** | **Chl *a*** | **Syn** | **PEUK** | **HB** | **NP** |
| --- | --- | --- | --- | --- | --- | --- | --- | --- | --- | --- | --- | --- | --- | --- |
| **NP** | **-0.58^**^** | -0.17 | 0.13 | **-0.49^**^** | **0.33^*^** | 0.21 | -0.20 | 0.21 | -0.28 | **0.41^**^** | **0.44^**^** | 0.25 | **0.49^**^** | 1.00 |
| **HNP** | **-0.48^**^** | 0.01 | **0.33^*^** | -0.22 | 0.08 | 0.09 | **-0.29^*^** | -0.01 | -0.21 | 0.10 | **0.37^**^** | 0.09 | **0.50^**^** | **0.85^**^** |
| **PNP** | **-0.39^**^** | **-0.41^**^** | -0.21 | **-0.61^**^** | **0.46^**^** | 0.25 | 0.00 | **0.38^**^** | **-0.31^*^** | **0.65^**^** | **0.29^*^** | **0.30^*^** | 0.243 | **0.73^**^** |
| **size_2_5** | **-0.55^**^** | -0.21 | 0.08 | **-0.43^**^** | **0.35^*^** | 0.22 | -0.17 | 0.18 | -0.23 | **0.39^**^** | **0.42^**^** | **0.28^*^** | **0.40^**^** | **0.94^**^** |
| **size_5_10** | **-0.45^**^** | -0.18 | 0.16 | **-0.45^**^** | 0.19 | 0.14 | -0.07 | 0.18 | -0.21 | **0.38^**^** | **0.39^**^** | 0.16 | **0.41^**^** | **0.62^**^** |
| **size_10_20** | -0.02 | 0.19 | **0.40^**^** | 0.03 | -0.22 | -0.08 | -0.16 | -0.09 | -0.03 | -0.13 | 0.08 | **-0.37^**^** | **0.37^**^** | **0.33^*^** |
| **Chao1** | 0.10 | **0.34^**^** | **0.50^**^** | **0.55^**^** | **-0.37^**^** | **-0.42^**^** | -0.07 | **-0.35^**^** | 0.21 | **-0.49^**^** | 0.00 | -0.19 | 0.07 | **-0.39^**^** |
| **Shannon** | -0.04 | 0.24 | **0.65^**^** | **0.46^**^** | **-0.33^*^** | -0.17 | -0.04 | **-0.26^*^** | 0.21 | **-0.38^**^** | 0.04 | -0.16 | 0.14 | -0.27 |
| **Pielou’s** | -0.11 | 0.16 | **0.65^**^** | **0.38^**^** | **-0.28^*^** | -0.07 | -0.01 | -0.18 | 0.20 | **-0.30^*^** | 0.07 | -0.11 | 0.16 | -0.20 |
| **. Correlation is significant at the 0.01 level (2-tailed). *. Correlation is significant at the 0.05 level (2-tailed). | | | | | | | | | | | | | | |

**Supplementary Table S6** Mantel tests for the correlations among spatial distance "S", environmental distance "E" and temporal factors' distance "T" in all samples, coast and shelf area, surface and bottom layer, as well as three months using Spearman's rank coefficients. Data in bold indicate significant correlations, * *P* < 0.05, ** *P* < 0.01.

| **Simple**  **mantel tests** | **E vs. T** | |  | **E vs. S** | |  | **S vs. T** | |
| --- | --- | --- | --- | --- | --- | --- | --- | --- |
|  | **r** | ***P*** |  | **r** | ***P*** |  | **r** | ***P*** |
| **All samples** | **0.130** | **0.003**** |  | -0.099 | 0.900 |  | -0.057 | 0.959 |
| **Coast** | **0.333** | **0.002**** |  | -0.048 | 0.654 |  | -0.095 | 0.987 |
| **Shelf** | -0.024 | 0.614 |  | 0.020 | 0.378 |  | -0.092 | 0.981 |
| **Surface** | **0.131** | **0.029*** |  | -0.103 | 0.803 |  | -0.093 | 0.967 |
| **Bottom** | **0.210** | **0.005**** |  | -0.156 | 0.895 |  | -0.093 | 0.951 |
| **May** | NA | NA |  | 0.027 | 0.405 |  | NA | NA |
| **August** | NA | NA |  | -0.159 | 0.932 |  | NA | NA |
| **October** | NA | NA |  | 0.176 | 0.067 |  | NA | NA |
